# The alarmone (p)ppGpp confers tolerance to oxidative stress during the stationary phase by maintenance of redox and iron homeostasis in *Staphylococcus aureus*

**DOI:** 10.1016/j.freeradbiomed.2020.10.322

**Published:** 2020-12

**Authors:** Verena Nadin Fritsch, Vu Van Loi, Tobias Busche, Quach Ngoc Tung, Roland Lill, Petra Horvatek, Christiane Wolz, Jörn Kalinowski, Haike Antelmann

**Affiliations:** a Freie Universität Berlin, Institute of Biology-Microbiology, D-14195, Berlin, Germany; b Center for Biotechnology, Bielefeld University, D-33594, Bielefeld, Germany; c Institute of Cytobiology, Philipps-University of Marburg, D-35037, Marburg, Germany; d Research Center for Synthetic Microbiology SynMikro, Hans-Meerwein-Str., D-35043, Marburg, Germany; e Interfaculty Institute of Microbiology and Infection Medicine, University of Tübingen, D-72076, Tübingen, Germany

**Keywords:** *Staphylococcus aureus*, (p)ppGpp, Stringent response, ROS, HOCl, Antibiotics

## Abstract

Slow growing stationary phase bacteria are often tolerant to multiple stressors and antimicrobials. Here, we show that the pathogen *Staphylococcus aureus* develops a non-specific tolerance towards oxidative stress during the stationary phase, which is mediated by the nucleotide second messenger (p)ppGpp. The (p)ppGpp^0^ mutant was highly susceptible to HOCl stress during the stationary phase. Transcriptome analysis of the (p)ppGpp^0^ mutant revealed an increased expression of the PerR, SigB, QsrR, CtsR and HrcA regulons during the stationary phase, indicating an oxidative stress response. The (p)ppGpp^0^ mutant showed a slight oxidative shift in the bacillithiol (BSH) redox potential (*E*_BSH_) and an impaired H_2_O_2_ detoxification due to higher endogenous ROS levels. The increased ROS levels in the (p)ppGpp^0^ mutant were shown to be caused by higher respiratory chain activity and elevated total and free iron levels. Consistent with these results, N-acetyl cysteine and the iron-chelator dipyridyl improved the growth and survival of the (p)ppGpp^0^ mutant under oxidative stress. Elevated free iron levels caused 8 to 31-fold increased transcription of Fe-storage proteins ferritin (*ftnA*) and miniferritin (*dps*) in the (p)ppGpp^0^ mutant, while Fur-regulated uptake systems for iron, heme or siderophores (*efeOBU*, *isdABCDEFG*, *sirABC* and *sstADBCD*) were repressed. Finally, the susceptibility of the (p)ppGpp^0^ mutant towards the bactericidal action of the antibiotics ciprofloxacin and tetracycline was abrogated with N-acetyl cysteine and dipyridyl. Taken together, (p)ppGpp confers tolerance to ROS and antibiotics by down-regulation of respiratory chain activity and free iron levels, lowering ROS formation to ensure redox homeostasis in *S. aureus*.

## Highlights

• High (p)ppGpp levels mediate tolerance to HOCl and MHQ stress in *S. aureus* during the stationary phase.
• High (p)ppGpp levels repress aerobic respiration and free iron levels to lower ROS formation.
• Decreased ROS lead to more reduced *E*_BSH_ promoting tolerance to oxidants and antibiotics in *S. aureus*.
• (p)ppGpp is required to maintain redox and iron homeostasis during the stationary phase.

## Graphical abstract

**Figure:**
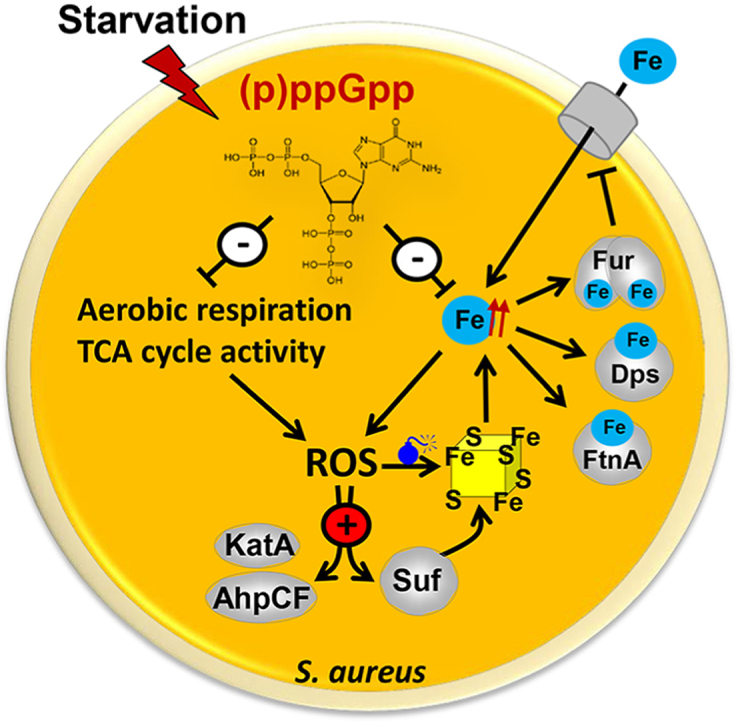

## 1 Introduction

*Staphylococcus aureus* is an opportunistic pathogen, which colonizes the nose and the skin of one quarter of the human population, but can also cause severe life-threatening infections [[1], [2], [3], [4], [5]]. The success of *S. aureus* as major human pathogen is further caused by the increasing prevalence of multiple antibiotic-resistant strains with limited treatment options, such as methicillin-resistant *S. aureus* isolates (MRSA) [6,7]*.* During acute and chronic infections, *S. aureus* has to combat with the oxidative burst of the host innate immune defense. Activated macrophages and neutrophils produce large amounts of reactive oxygen and chlorine species (ROS, RCS), such as H_2_O_2_ and HOCl as the first line defense to kill invading pathogens [[8], [9], [10], [11]]. In addition, *S. aureus* has to adapt to antimicrobial compounds and reactive electrophilic species (RES), such as quinones during host-pathogen interactions. Thus, it is of utmost importance to study the defense and resistance mechanisms of *S. aureus* under ROS, RCS, RES and antibiotics for identification of new drug targets and development of alternative therapy strategies to combat infections with multi-resistant *S. aureus* isolates [12].

During infections, *S. aureus* produces an arsenal of different virulence factors, such as toxins and extracellular enzymes that are secreted during the stationary phase to damage host tissues [13]. In addition, *S. aureus* encodes several stressor-specific defense mechanisms to cope with ROS, RCS, RES and antibiotics treatment [10,[14], [15], [16]]. The low molecular weight thiol bacillithiol (BSH) and its associated bacilliredoxin (Brx)/BSH/bacillithiol disulfide reductase (YpdA) pathway play important roles to maintain redox homeostasis during recovery from oxidative stress [[17], [18], [19]]. Moreover, several redox regulators, including PerR, HypR, MgrA, SarZ, QsrR and MhqR sense ROS, RCS and RES to control specific detoxification pathways for degradation of redox-active compounds or to repair the resulting damage in *S. aureus* [17,18,[20], [21], [22], [23]]. Such mechanisms provide protection against the respective reactive species and contribute to virulence and survival of the pathogen.

Apart from specific stress responses, many bacteria acquire a non-specific prospective resistance to multiple stressors and antibiotics during the stationary phase, which can be provoked by nutrient starvation, physical and chemical stressors [24,25]. In *Bacillus subtilis*, the alternative sigma factor SigmaB was shown to control a large general stress and starvation regulon which confers resistance and cross-protection to multiple stimuli, such as heat, salt and oxidative stress during the stationary phase [26]. However, the mechanisms of starvation-induced stationary phase resistance to stress and antibiotics are not fully understood in *S. aureus*.

In bacteria, the small alarmone (p)ppGpp accumulates during entry into the stationary phase by amino acid or carbon source limitation leading to the stringent response (SR) [[26], [27], [28], [29]]. The SR is characterized by down-regulation of processes required for active growth, such as cell division, replication, transcription and translation, mediated by the repression of genes for rRNAs, ribosomal proteins and translation factors [30]. The main goal of the SR is to save energy and cellular resources during the non-growing state [29,31]. In addition, stress defense mechanisms and amino acid biosynthesis pathways are induced under SR conditions to ensure continued synthesis of stress proteins that are required for bacterial survival [24,25]. In *S. aureus*, the bifunctional synthase/hydrolase Rel (RelA/SpoT homolog) and two truncated (p)ppGpp synthases (RelP and RelQ) catalyze the pyrophosphate transfer from ATP to GTP or GDP to synthesize (p)ppGpp [[32], [33], [34], [35]]. Compared to the many targets discovered for (p)ppGpp in Gram-negative bacteria, little is known about (p)ppGpp targets in Gram-positive firmicutes. In many bacteria, GTPases can be competitively inhibited by (p)ppGpp, including the ribosomal translation factors EF-Tu, EF-G, RF3 and IF2 [28,[36], [37], [38], [39]]. In *S. aureus*, (p)ppGpp was shown to inhibit two enzymes needed for GTP synthesis (HprT and Gmk) and five GTPases (RsgA, RbgA, Era, HflX and ObgE) that are implicated in ribosome assembly [40,41]. The lack of GTP synthesis leads to inhibition of transcription of ribosomal RNAs that require GTP as initiating NTP [42,43]. In firmicutes, the decreased GTP pool upon (p)ppGpp synthesis causes inactivation of the CodY repressor, resulting in derepression of amino acid biosynthesis genes as part of the SR [40].

In addition, the SR is associated with virulence, biofilm formation, persister formation and involved in stationary phase-induced antibiotics tolerance [[44], [45], [46], [47], [48], [49], [50]]. The *S. aureus* (p)ppGpp^0^ mutant which lacks all three (p)ppGpp synthases showed increased sensitivity to cell wall-active antibiotics, such as vancomycin and ampicillin and was impaired in survival in phagocytosis assay [51,52]. In addition, (p)ppGpp conferred high level of beta lactam resistance in MRSA strains *via* increased expression of penicillin-binding proteins, encoded by *mecA* and *pbpD* [47,48]. Overproduction of (p)ppGpp due to *rel* mutations in clinical isolates resulted in increased tolerance to five different antibiotic classes [53].

In *Vibrio cholerae*, (p)ppGpp was shown to reduce endogenous ROS formation possibly by inhibition of the iron-uptake transporter FbpA, which promotes tolerance to the antibiotic tetracycline [54]. Furthermore, (p)ppGpp down-regulates TCA cycle enzymes of central carbon metabolism and aerobic respiration to decrease ROS levels [54]. The *Pseudomonas aeruginosa* SR mutant suffered from increased ROS levels due to reduced activities of catalases and superoxide dismutases resulting in decreased multidrug tolerance [[54], [55], [56]]. Thus, several studies provide a link between (p)ppGpp and increased antibiotic tolerance via ROS levels. Moreover, ROS were shown to be involved in the killing mode of different antibiotic classes, which involves cellular respiration, metabolic pathways and the redox state [[57], [58], [59]]. Thus, factors that regulate the redox balance of bacteria play an important role in virulence and antibiotic susceptibility.

In this study, we found that *S. aureus* acquires a non-specific resistance towards oxidative stress during the stationary phase, which was dependent on the SR mediated by (p)ppGpp. We therefore investigated the mechanisms of underlying ROS susceptibility in the (p)ppGpp^0^ mutant. Expression of the antioxidant stress response and iron-storage ferritins was induced in the (p)ppGpp^0^ mutant during the stationary phase due to elevated ROS and free iron levels leading to decreased tolerance to HOCl, tetracycline and ciprofloxacin. In addition, higher respiratory chain activity contributed to ROS increase in the absence of (p)ppGpp. Thus, (p)ppGpp impacts aerobic respiration, iron and redox homeostasis in *S. aureus* to promote tolerance to antibiotics and oxidative stress during the stationary phase, which could be particularly important during long-term and chronic infections with MRSA strains.

## 2 Materials and methods

### 2.1 Bacterial strains, growth and survival assays

Bacterial strains and primers are listed in Tables S1 and S2. The *S. aureus* strains used in this study were *S. aureus* COL and USA300JE2 wild types (WT) and the USA300JE2 derivative with mutations in the *rel* synthetase domain (Δ*rel*_*syn*_*)*, which was transduced from the restriction-negative intermediate RN4220 Δ*rel*_*syn*_ into strain USA300JE2 as previously described [52]. The USA300JE2 (p)ppGpp^0^ strain contained mutations in the active sites of each of the three (p)ppGpp synthetases, *relP, relQ* and *rel* [60]. The (p)ppGpp^0^ strain was complemented with plasmid pCG327, which expresses the Rel synthetase (Rel_syn_) under the control of an anhydrotetracycline (AHT) inducible promotor [61] (Table S1). The Brx-roGFP2 biosensor expressing strains USA300JE2 pRB473-*brx-roGFP2* and USA300JE2 (p)ppGpp^0^ pRB473-*brx-roGFP2* were constructed by phage transduction from RN4220 pRB473-*brx-roGFP2* into USA300JE2 and the isogenic (p)ppGpp^0^ mutant as previously described [62]. Construction of the *katA* mutant was described previously [63]. For growth and survival assays, *S. aureus* strains were cultivated in RPMI 1640 cell culture medium (Bioscience Lonza, Catalog No. BE12-918F) containing 0.75 μM FeCl_3_. Survival was determined by plating 100 μl of serial dilutions of *S. aureus* strains after 1–2 h of stress exposure onto LB agar plates for CFUs counting. Brx-roGFP2 biosensor measurements were conducted by cultivation of *S. aureus* WT and (p)ppGpp^0^ mutant strains with plasmid pRB473-*brx-roGFP2* in LB and Belitsky minimal medium as described previously [62,64]. Statistical analysis was performed using the Student's unpaired two-tailed *t*-test by the graph prism software. The chemicals and antibiotics methylhydroquinone (MHQ), NaOCl, 2,2′−dipyridyl, N-acetyl cysteine, FeCl_3,_ ciprofloxacin, tetracycline and streptonigrin were purchased from Sigma Aldrich and Merck, respectively. NaOCl dissociates in aqueous solution to hypochlorous acid (HOCl) and hypochlorite (OCl^−^) [65]. Thus, the concentration of HOCl was determined by absorbance measurements as reported previously [66].

### 2.2 RNA isolation, northern blot analysis, transcriptome sequencing and bioinformatics

For RNA isolation, *S. aureus* strains were cultivated in RPMI and LB medium and harvested during the log and stationary phases as indicated in the figure and table legends. Northern blot hybridizations were performed as described [67,68] with the digoxigenin-labeled antisense RNA probes specific for the transcripts RNAIII, *katA*, *ahpC*, *ftnA*, *dps, ohr*, *clpB* and *asp23,* which were synthesized *in vitro* using T7 RNA polymerase and the specific primer pairs as described previously [20,69] and in Table S2.

Transcriptome sequencing was performed using RNA of *S. aureus* USA300JE2 and the (p)ppGpp^0^ mutant grown in RPMI medium and harvested at an OD_500_ of 0.5 and 1.2 for log and stationary phases, respectively, as described [21]. Differential gene expression analysis of 3 biological replicates was performed using DESeq2 [70] with ReadXplorer v2.2 [71] as described previously [21] using an adjusted *p*-value cutoff of ≤0.05 and a signal intensity ratio (*M*-value) cutoff of ≥0.6 or ≤ −0.6 (fold-change of ±1.5). Genes were sorted into regulons based on the RegPrecise database as in previous studies [21]. Whole transcriptome RNA-seq raw data files are available in the ArrayExpress database under accession number E-MTAB-9368.

### 2.3 Determination of intracellular iron levels using ferene-s assay and ICP-mass spectrometry

The intracellular iron concentrations of *S. aureus* USA300JE2 WT, (p)ppGpp^0^ and *rel*_*syn*_ mutants as well as the complemented (p)ppGpp^0^ pCG327 strain were determined with ferene-s (3-(2-pyridyl)-5,6-di (2-furyl)-1,2,4-triazine-5′,5″-disulfonic acid disodium salt) assay purchased from Sigma-Aldrich according to the instructions of the manufacturer with some modifications. In brief, *S. aureus* strains grown in RPMI were harvested during the log and stationary phases at an OD_500_ of 0.5, 1 and 2, respectively. Cell pellets were lysed with 1% hydrochloric acid (HCl) and heated at 80 °C for 10 min. Excess acid was neutralized with 7.5% ammonium acetate. Next, ferric iron (Fe^3+^) was reduced to ferrous iron (Fe^2+^) with 4% ascorbic acid. Precipitated protein was complexed with 2.5% sodium dodecyl sulfate. About 1.5% of the iron chelator ferene-s was added leading to the formation of a blue iron-ferene-s complex (Fe^2+^: ferene-s). Samples were centrifuged at 9000 rpm for 7 min and the absorbance was measured at 593 nm. Ammonium iron (II) sulfate hexahydrate (Sigma Aldrich) was used to prepare the iron standard curve.

Iron levels were further determined using inductively coupled plasma (ICP) mass spectrometry. *S. aureus* cell cultures of ~70–280 ml, containing total protein amounts of ~6–10 mg were harvested by centrifugation. The dried cell pellets were mixed with an excess of concentrated nitric acid (69%) and incubated at 60 °C for at least 2 h to destroy any organic content. Samples were diluted with water to a final nitric acid concentration of 10%. For metal detection, samples were further diluted 1:10, and 1 ppb rhodium was added as an internal standard. Elements of interest were quantified using an Element 2 ICP-MS system (Thermo Scientific™, Bremen). For ionization of analytes, a plasma was generated with a power of 1200 W. For quantitation, the standard addition method was utilized. A matrix sample was used as blank and subtracted.

### 2.4 Measurements of BSH redox potential (*E*_BSH_) changes using the Brx-roGFP2 biosensor

*S. aureus* USA300JE2 WT and (p)ppGpp^0^ mutant strains expressing the Brx-roGFP2 biosensor were cultivated in LB and used for measurements of the biosensor oxidation degree (OxD) along the growth curves and after injection of H_2_O_2_ and HOCl into the microplate well as described [62,64]. Fully reduced and oxidized controls were prepared with 10 mM DTT and 20 mM cumene hydroperoxide, respectively. Brx-roGFP2 biosensor fluorescence emission was measured at 510 nm after excitation at 405 and 488 nm using the CLARIOstar microplate reader (BMG Labtech). The OxD of the Brx-roGFP2 biosensor was determined for each sample and normalized to fully reduced and oxidized controls. Based on the OxD and $E_{roGFP2}^{0^{\prime }}$ = - 280 mV [72], the BSH redox potential (*E*_BSH_) was calculated according to the Nernst equation [62]. The *E*_BSH_ results are presented in Table S3.

### 2.5 FOX assay for determination of H_2_O_2_ detoxification capacity of cell extracts

The FOX assay was used to determine the H_2_O_2_ consumption capacity of cytoplasmic extracts of *S. aureus* USA300JE2 WT, (p)ppGpp^0^ mutant and complemented (p)ppGpp^0^ pCG327 cells, which were harvested in RPMI at OD_500_ of 1.2 as described previously [73]. FOX reagent was prepared by adding 100 ml FOX I (100 mM sorbitol, 125 μM xylenol orange) to 1 ml FOX II (25 mM ammonium ferrous (II)sulfate in 2.5 M H_2_SO_4_). To prepare cytoplasmic extracts, cells were washed twice with 83 mM phosphate buffer (pH 7.05) and disrupted using the ribolyzer. Next, 100 μl cell lysate containing 10 μg protein was added to 500 μl of 10 mM H_2_O_2_ solution. After different times (1–5 min), 2 μl of the samples were added to 200 μl FOX reagent and incubated for 30 min at room temperature. The absorbance was measured at 560 nm using the CLARIOstar microplate reader. H_2_O_2_ standard curves were measured with 20 μl H_2_O_2_ (0–18 μM final concentrations) and 200 μl FOX reagent as above.

### 2.6 ROS measurements using 2′,7′-dichlorodihydrofluorescein diacetate (DCFH_2_-DA)

Endogenous ROS levels were measured using the 2′,7′-dichlorodihydrofluorescein diacetate (DCFH_2_-DA) dye (Th. Geyer) [74]. DCFH_2_-DA is de-acetylated by alkaline hydrolysis by NaOH to generate DCFH_2_, which is oxidized by ROS to the fluorescent dye 2′,7′-dichlorofluorescein (DCF) using the previous protocol [75]. Briefly, *S. aureus* USA300JE2 wild type, the (p)ppGpp^0^, *rel*_*syn*_ and *katA* mutants as well as the complemented (p)ppGpp^0^ pCG327 strain were cultivated in RPMI medium to an OD_500_ of 0.5, 1 and 2. The cells were harvested at an OD_600_ equivalent of 5 × 10^8^ cells by centrifugation. Cell pellets were incubated with DCFH_2_ for 40 min as described [75]. Relative DCF fluorescence was measured using the CLARIOstar microplate reader at an excitation and emission wavelength of 488 and 515 nm, respectively.

### 2.7 Determination of catalase activity using native PAGE and diaminobenzidine staining

*S. aureus* strains were grown in RPMI and cytoplasmic extracts prepared as above for the FOX assay. Cytoplasmic extracts were separated using native PAGE and stained for catalase activity using the diaminobenzidine staining method as described previously [76,77].

### 2.8 Determination of oxygen consumption rates

For measurements of respiratory chain activity by oxygen consumption rates in *S. aureus* strains, the Clark-type electrode (Oxygraph, Hansatech) was used as described previously [21,78,79]. In brief, *S. aureus* strains were grown in RPMI to an OD_500_ of 0.5 and 1 or in TSB to an OD_600_ of 0.6 and 3. Cells were harvested by centrifugation, washed in 33 mM potassium phosphate buffer (pH 7.0) and adjusted to an OD_578_ of 5. Oxygen consumption rates were determined after addition of 1 mM glucose as electron donor in 3 biological replicates. The values were corrected for basal oxygen consumption without electron donors.

## 3 Results

### 3.1 *S. aureus* acquires a non-specific tolerance to oxidative stress during the stationary phase, which is mediated by the alarmone (p)ppGpp

**Fig. 1:**
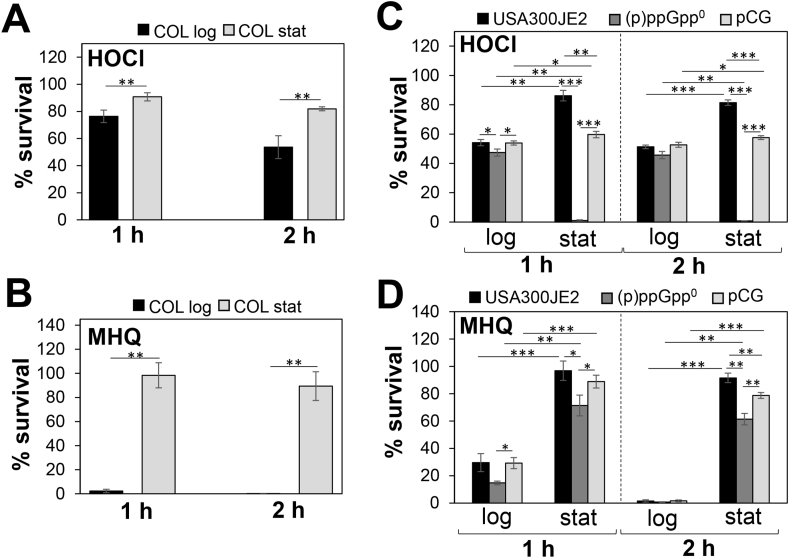
**The alarmone (p)ppGpp confers tolerance to oxidative stress in *S. aureus* during the stationary phase**. For survival assays, *S. aureus* COL and USA300JE2 wild type, the (p)ppGpp^0^ mutant and complemented strain (pCG) were exposed to 3.5 mM HOCl **(A,C)** and 400 μM MHQ **(B,D)** during the log and stationary phases at OD_500_ of 0.5 and 2–3, respectively. The CFUs after 1 and 2 h stress exposure were calculated as survival rates relative to the untreated control, which was set to 100%. The (p)ppGpp^0^ mutant was more sensitive to HOCl and MHQ stress, and impaired to acquire the stationary phase-induced tolerance towards HOCl. The results are from four biological replicates. Error bars represent the standard deviation. *p < 0.05; **p < 0.01; ***p < 0.001.

Previously, we characterized various stressor-specific resistance mechanisms that conferred protection of *S. aureus* to the thiol-reactive compounds HOCl and quinones during the exponential growth, including the redox-sensing HypR and MhqR repressors [18,21]. In this study, we were interested if *S. aureus* is able to develop non-specific tolerance to HOCl and MHQ during the stationary phase. Thus, survival rates of the *S. aureus* COL and USA300JE2 isolates were determined after exposure to 3.5 mM HOCl and 400 μM MHQ during the log and stationary phases at OD_500_ of 0.5 and 2–3, respectively (Fig. 1). These doses reduced the survival of log phase bacteria, but stationary phase cells displayed an enhanced survival rate upon HOCl and MHQ treatment (Fig. 1). Specifically, the survival of stationary phase cells was 40-fold increased after 400 μM MHQ treatment compared to log phase cells, while only <2-fold elevated survival rates were determined in stationary phase cells in response to 3.5 mM HOCl challenge (Fig. 1).

Next, we analyzed the survival of the USA300JE2 (p)ppGpp^0^ mutant, which cannot synthesize (p)ppGpp, after exposure to 3.5 mM HOCl and 400 μM MHQ during the log or stationary phases (Fig. 1C and D). While the survival of HOCl-treated log phase cells of the (p)ppGpp^0^ mutant was only slightly different from the WT, strong killing of mutant cells was observed with only 2% survivors during the stationary phase (Fig. 1C). In contrast, the absence of (p)ppGpp resulted in a similar enhanced stationary phase-induced tolerance to MHQ stress compared to the WT (Fig. 1D). However, the (p)ppGpp^0^ mutant was more sensitive to MHQ treatment during the log and stationary phases relative to its parent strain. Plasmid-borne expression of the (p)ppGpp synthetase Rel_syn_ (pCG327) restored the stationary phase tolerance of the (p)ppGpp^0^ mutant under HOCl and MHQ stress (Fig. 1C and D). These results indicate that (p)ppGpp protects from oxidative and quinone stress provoked by HOCl and MHQ during the log and stationary phases.

### 3.2 The absence of (p)ppGpp induces an oxidative and iron stress response in *S. aureus* during the stationary phase in the transcriptome

**Fig. 2:**
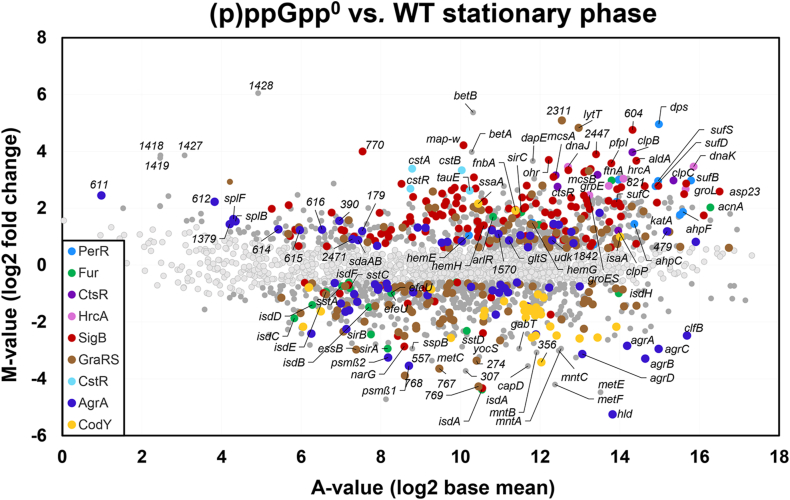
**Transcriptome analysis reveals an oxidative and iron stress response in the (p)ppGpp**^**0**^**mutant during the stationary phase**. *S. aureus* USA300JE2 and the (p)ppGpp^0^ mutant were grown in RPMI medium and RNA was isolated from cells harvested at an OD_500_ of 1.2. The gene expression profile of the (p)ppGpp^0^ mutant versus the WT is shown as ratio/intensity scatterplot (M/A-plot), which is based on the differential gene expression analysis using DeSeq2. Colored symbols indicate significantly induced and repressed transcripts (M-value ≥0.6 or ≤ −0.6; *p* ≤ 0.05), which could be allocated to the PerR (blue), Fur (green), CtsR (magenta), HrcA (pink), SigB (red), GraRS (brown), CstR (light blue), Agr (dark blue) and CodY regulons (yellow). Light gray symbols denote transcripts with no fold-changes (*p* > 0.05). The complete transcriptome data and regulon classifications are listed in Tables S4–S7. (For interpretation of the references to colour in this figure legend, the reader is referred to the Web version of this article.)

To understand the mechanisms of impaired stationary phase tolerance to HOCl stress in the (p)ppGpp^0^ mutant, we analyzed the gene expression changes in the (p)ppGpp^0^ mutant versus WT cells during the log and stationary phases using transcriptomics (Fig. 2, S1-S2, Tables S4–S9). Significant changes were determined by an M-value cut-off (log2-fold change (p)ppGpp^0^ mutant/WT, *p* ≤ 0.05) of ≥0.6 and ≤-0.6 (fold-change of ± 1.5, P ≤ 0.05). In total, 282 and 190 genes were significantly >1.5-fold up- and down-regulated, respectively in the (p)ppGpp^0^ mutant compared to the WT during the stationary phase (Fig. 2, Tables S4–S9). The most interesting stress and starvation-induced or repressed regulons in the (p)ppGpp^0^ mutant are labeled in the ratio/intensity scatter plots (M/A-plots) (Fig. 2, S1-S2, Tables S4–S6).

Among the top scorers are the oxidative stress responsive PerR, CstR, QsrR, CtsR and HrcA regulons, which were highly elevated in the (p)ppGpp^0^ mutant during the stationary phase (Fig. 2, Tables S4–S6). However, this oxidative stress response was not induced in log phase cells (Fig. S2, Tables S7–S9). The peroxide sensing PerR repressor controls genes for H_2_O_2_ detoxification, heme and iron sulfur cluster biogenesis [80], including the catalase *katA* (3.4-fold induced), the peroxidase *ahpCF* (2.7–3.6-fold), the miniferritin *dps* (31-fold), the *hemEHY* operon (2–2.8-fold) and the *sufCDSUB* operon (4.7-8-fold) (Fig. 2, Tables S4–S6). The induction of the oxidative stress response could point to increased ROS levels in the absence of (p)ppGpp. In addition, the genes and operons of the CtsR and HrcA regulons for proteases and chaperones displayed the highest fold-changes, including *clpB* (15.6-fold), *clpP* (2.3-fold), *ctsR-mcsA-mcsB-clpC* (6.8-9-fold), *hrcA-grpE-dnaKJ* (6.9–11-fold) and *groESL* (5.5–6.4-fold). These protein quality control machineries facilitate protein folding and degradation of oxidatively damaged proteins and are associated with the thiol stress response as shown previously under HOCl, AGXX® and allicin stress [18,21,[81], [82], [83], [84], [85]].

Of note, the transcriptome results further revealed a very strong iron stress response in the (p)ppGpp^0^ mutant since Fe-storage ferritin (*ftnA)* and miniferritin (*dps)* were most strongly 8-31-fold up-regulated [23,80,86] (Fig. 2, Tables S4–S6). Elevated iron storage is further supported by the increased transcription of *sufCDSUB* operon for enhanced iron-sulfur cluster biosynthesis, suggesting increased iron levels in the (p)ppGpp^0^ mutant. In contrast, the majority of Fur-controlled uptake systems for iron, heme and siderophores were strongly down-regulated in the (p)ppGpp^0^ mutant. The repressed iron transporters include the iron-dependent peroxidase *efeOBU* operon (0.5–0.7-fold)*,* the Fe(III)-staphyloferrin-B *sirAB* uptake system (0.13–0.18-fold), the catechol-type Fe(III) siderophore importer *sstABC* operons and the iron‐regulated surface determinant *isdABCDEFG* operon for uptake of heme iron (0.05–0.5-fold) [87,88]. In addition, down-regulation of the Mn(II) uptake *mntABC* operon (0.12–0.13-fold) further denotes metal ion dysregulation in the (p)ppGpp^0^ mutant. Thus, the up-regulation of the PerR regulon, the increased iron-storage and the repressed iron transport together support the hypothesis of a prevailing internal iron excess, which may lead to ROS formation via Fenton chemistry [54,80].

Moreover, the sulfur-stress specific CstR regulon (6.1–10.5-fold) and the quinone stress responsive QsrR regulon (2.5–5.3-fold) were both strongly upregulated in the mutant. The CstR and QsrR repressors harbour redox-sensing Cys residues and were previously shown to respond to disulfide stress, such as HOCl, AGXX® and allicin stress in *S. aureus* [18,22,[84], [85], [86],^89^]*.* Additionally, genes for cysteine and BSH biosynthesis (*cysK, bshB, bshC*) displayed 2-4-fold higher expression levels in the (p)ppGpp^0^ mutant, indicating an impaired redox homeostasis in the (p)ppGpp^0^ mutant. Furthermore, the majority of genes of the SigB general stress regulon were strongly up-regulated in the (p)ppGpp^0^ mutant during the stationary phase (Fig. 2, Tables S4–S6). These include the *sigB-rsbW-rsbV-rsbU*-operon (3.6–4.1-fold), the *asp23-SACOL2174-amaP-opuD2* operon (6–9.7-fold), the multi-drug transporter *bmrU* (10.1-fold), the capsule biosynthesis *cap* operon (2-4-fold), the staphyloxanthin biosynthesis *crtNMQIO* operon (2.3–6.5-fold), the aldehyde dehydrogenase *aldA* (12.7-fold), the *ohr* peroxiredoxin (13-fold) and several genes that code for hypothetical proteins.

In addition, the cell wall stress-responsive GraRS regulon [90,91] was partially up- or down-regulated in the (p)ppGpp^0^ mutant (Tables S4–S6). Among the GraRS regulon were highly induced the flavodoxin *acpD* gene (7-fold), the TCA cycle enzymes *citCZ* and *sucCD* operons (2-4-fold), the cytochrome D ubiquinol oxidase *cydAB* operon (4-6-fold), the glycine-betaine transporter *opuCA-CB-CC-CD* operon (3.2-4-fold) and the *SAUSA300_2310/2311* operon (28-34-fold) of unknown functions. Elevated expression of the *citCZ, sucCD and cydAB* operons could indicate an enhanced TCA cycle activity and higher respiratory chain activity in the (p)ppGpp^0^ mutant, which might contribute to ROS formation. Apart from the *opuC* operon, we found a strong up-regulation of the *betAB* operon that codes for a glycine betaine synthetase (15.8–41-fold), pointing to an enhanced synthesis and uptake of compatible solutes in the (p)ppGpp^0^ mutant.

As expected, the CodY regulon genes for amino acid biosynthesis were strongly down-regulated in the absence of (p)ppGpp due to increased GTP-levels resulting in stronger CodY repression [51]. The main role of (p)ppGpp is to downregulate genes for active growth and to stop protein translation [31]. Thus, the majority of genes encoding ribosomal proteins, translation elongation factors and aminoacyl-tRNA synthetases were 2-10-fold induced in the absence of (p)ppGpp (Fig. 2, S1, Tables S4–S9). Altogether, the transcriptome signature of the (p)ppGpp^0^ mutant supports the hypothesis of an oxidative and iron stress response due to enhanced respiration and iron overload leading to ROS production and an impaired redox balance.

### 3.3 The oxidative and iron stress response is elevated in the (p)ppGpp^0^ mutant only in RPMI medium during the stationary phase

**Fig. 3:**
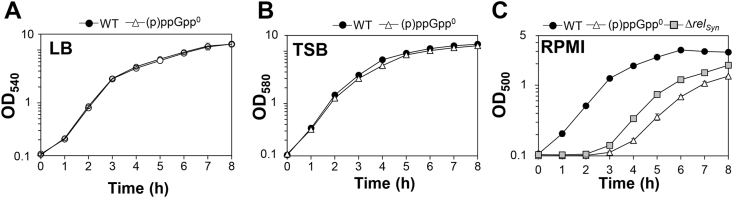
**The (p)ppGpp**^**0**^**mutant shows a growth defect in RPMI medium.** Growth curves were monitored of the *S. aureus* USA300JE2 wild type (WT), the (p)ppGpp^0^ and Δ*rel*_*syn*_ mutants in LB **(A)**, TSB **(B)** and RPMI medium **(C)**.

**Fig. 4:**
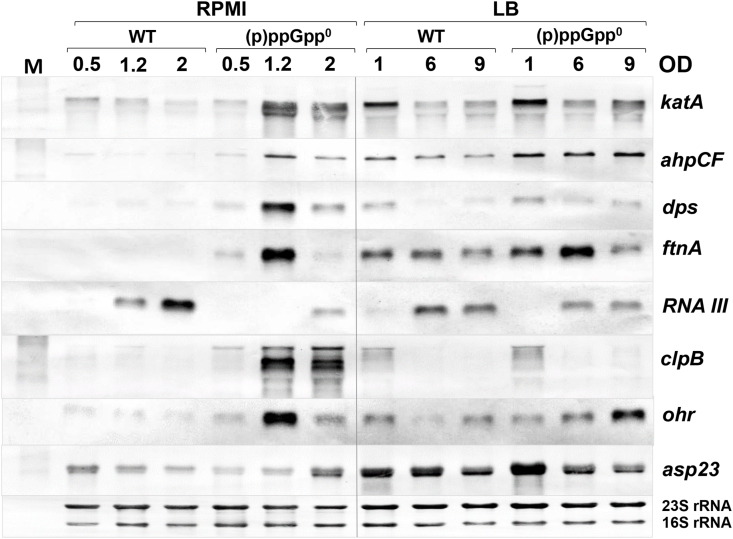
**The oxidative and iron stress response is elevated in the (p)ppGpp**^**0**^**mutant only in RPMI during the stationary phase.** Northern blot transcriptional analysis was performed for the PerR (*katA, ahpCF*), SigB (*asp23, ohr*), CtsR (*clpB*) and Agr (RNAIII) regulons and the iron storage ferritins (*dps, ftnA*) in *S. aureus* USA300JE2 WT and the (p)ppGpp^0^ mutant in RPMI and LB during the log and stationary phases. The (p)ppGpp^0^ mutant showed increased transcription of genes for iron storage (*dps*, *ftnA*), ROS detoxification (*katA, ahpCF*) and protein quality control (*clpB*) in RPMI. The methylene blue stain is the RNA loading control indicating the 16S and 23S rRNAs.

Previous studies revealed no growth defects of the (p)ppGpp^0^ mutant in complex LB or TSB medium [33], which was confirmed in our study (Fig. 3A and B). However, the (p)ppGpp^0^ and *rel*_*syn*_ mutants revealed a strong growth delay when cultivated in RPMI (Fig. 3C). Thus, we were interested to unravel the underlying mechanism of the growth delay of the (p)ppGpp^0^ mutant in RPMI medium. Based on the transcriptome data, the (p)ppGpp^0^ mutant showed an enhanced oxidative and iron stress response in RPMI during the stationary phase (Fig. 2, Tables S4–S6). Thus, we used Northern blots to investigate whether the oxidative and iron stress response is responsible for the growth defect of the (p)ppGpp^0^ mutant in RPMI during the stationary phase (Fig. 4). The Northern blot results of the (p)ppGpp^0^ mutant in LB did not reveal differences in transcription of oxidative stress genes controlled by PerR (*katA*, *ahpCF*, *dps*), CtsR (*clpB*), SigB (*asp23, ohr*) and Agr (RNAIII). Only slightly increased transcription in the (p)ppGpp^0^ mutant versus WT cells was observed for the peroxiredoxin (*ohr*) and the ferritin (*ftnA*) genes in LB during the stationary phase (Fig. 4). These data support that WT and (p)ppGpp^0^ mutant cells do not show growth differences in rich LB medium. In contrast, cultivation in RPMI medium resulted in up-regulation of genes encoding antioxidant enzymes (*katA*, *ahpCF, ohr*), the Clp protease (*clpB*) and iron storage proteins (*dps*, *ftnA*) in the (p)ppGpp^0^ mutant during the stationary phase compared to the WT (Fig. 4). This induction of the oxidative and iron stress responses in the (p)ppGpp^0^ mutant was abrogated in the (p)ppGpp complemented *rel*_*syn*_ strain (Fig. S3). Furthermore, transcription of the Agr-controlled RNAIII was down-regulated in the (p)ppGpp^0^ mutant in RPMI, which is consistent with the strong repression of the *agrABCD* operon in the transcriptome and might be related to the slower growth rate (Fig. 2, Fig. 4). Overall, these transcriptional results support the hypothesis that the (p)ppGpp^0^ mutant suffers from oxidative and iron stress in RPMI medium during the stationary phase, which might be responsible for its growth delay in RPMI (Fig. 3C).

### 3.4 The (p)ppGpp^0^ mutant shows a slight oxidative shift in the BSH redox potential, an increased ROS level and delayed H_2_O_2_ detoxification

**Fig. 5:**
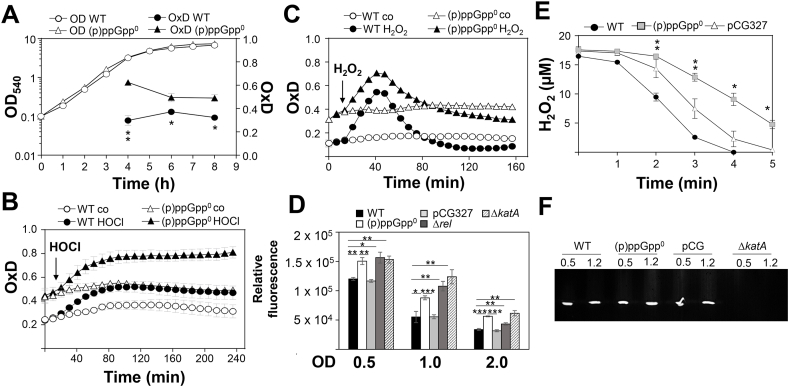
**The (p)ppGpp**^**0**^**mutant shows an oxidized basal BSH redox potential (*E***_**BSH**_**) (A, B, C), an elevated endogenous ROS level(D)and is delayed in H**_**2**_**O**_**2**_**detoxification (E). (A)** The basal level of *E*_BSH_ was measured in LB medium using the Brx-roGFP2 biosensor along the growth curve in *S. aureus* USA300JE2 WT and the (p)ppGpp^0^ mutant. **(B, C)** Oxidation of the Brx-roGFP2 biosensor was monitored in *S. aureus* USA300JE2 and the (p)ppGpp^0^ mutant after exposure to 150 μM HOCl **(B)** and 100 mM H_2_O_2_**(C)**. The (p)ppGpp^0^ mutant showed a slight oxidative shift of the basal *E*_BSH_, but is not impaired in the response to H_2_O_2_ and HOCl stress. The Brx-roGFP2 biosensor responses are shown as OxD values which were calculated based on 405/488 nm excitation ratios with emission at 510 nm and related to the fully oxidized and reduced controls. The *E*_BSH_ changes were calculated using the Nernst equation and presented in Table S3. **(D)** Intracellular ROS levels were quantified in the *S. aureus* USA300JE2 WT, the (p)ppGpp^0^ mutant, the complemented strain (pCG327), the *rel*_*syn*_ mutant and the *katA* mutant using the DCFH_2_-DA dye, which is oxidized to DCF by ROS. DCF fluorescence is measured after excitation at 488 nm and emission at 515 nm. **(E)** The FOX assay was used to determine the H_2_O_2_ detoxification ability in *S. aureus* WT, the (p)ppGpp^0^ mutant and the complemented strain (pCG327) during the stationary phase at OD_500_ of 1.2 in RPMI. H_2_O_2_ detoxification was slower in the (p)ppGpp^0^ mutant compared to the WT. The results are from 3 biological replicates. Error bars represent the standard deviation. *p < 0.05; **p < 0.01; ***p < 0.001. **(F)** The catalase activity was determined in cell extracts of *S. aureus* USA300JE2 WT, the (p)ppGpp^0^ mutant, the complemented strain (pCG) and the *katA* mutant during growth in RPMI using native PAGE and diamidobenzidine staining as described [76].

Transcriptional studies revealed an increased oxidative stress response in the absence of (p)ppGpp. Hence, we hypothesized that the (p)ppGpp^0^ mutant might have an impaired redox balance. The Brx-roGFP2 biosensor was applied to monitor the changes in the BSH redox potential (*E*_BSH_) inside WT and (p)ppGpp^0^ mutant cells during different growth phases and after oxidative stress (Fig. 5A–C). However, *E*_BSH_ changes could not be measured upon growth in RPMI medium due to low expression of the Brx-roGFP2 biosensor resulting in low fluorescence intensities, which did not allow ratiometric quantification of the fluorescence changes. Consequently, the oxidation degree (OxD) of the Brx-roGFP2 was measured along the growth in LB based on the ratiometric changes of the 405 nm and 488 nm excitation maxima upon roGFP2 oxidation as described previously (Fig. 5A) [62,64]. The corresponding *E*_BSH_ values were calculated from the OxD using the Nernst equation (Table S3). The biosensor results showed that WT cells maintained a highly reduced *E*_BSH_ of ~ -290 mV with little fluctuations during the log and stationary phases. However, a slight but significant oxidative shift in *E*_*B*SH_ to ~ -280 mV was determined throughout the growth in the (p)ppGpp^0^ mutant (Fig. 5A, Table S3). This higher basal level oxidation of Brx-roGFP2 in the (p)ppGpp^0^ mutant was most evident at an OD_540_ of 3 with an increased OxD of 0.63 compared to 0.3 in the WT (Fig. 5A). This oxidative shift of the basal OxD could be verified in the oxidant injection assays before treatment with H_2_O_2_ and HOCl, supporting an impaired basal redox state of the (p)ppGpp^0^ mutant (Fig. 5B and C). However, the biosensor response to 150 μM HOCl and 100 mM H_2_O_2_ was similar in both WT and (p)ppGpp^0^ mutant cells. While both strains were able to regenerate the reduced *E*_BSH_ within 80 min during recovery from H_2_O_2_ stress, regeneration of reduced *E*_BSH_ was not possible after HOCl stress (Fig. 5B and C). Thus, the (p)ppGpp^0^ mutant showed a slight oxidative shift in its basal *E*_BSH_ observed during the transition to stationary phase when grown in LB.

Next, we used the previously established DCFH_2_-DA assay to determine endogenous ROS levels in the *S. aureus* strains grown in RPMI medium [75]. The results showed an ~1.3-2-fold elevated fluorescence of the oxidized DCF dye in the (p)ppGpp^0^ and *rel*_*syn*_ mutants during the log and stationary phases as detected by excitation at 488 nm and emission at 515 nm (Fig. 5D). Thus, ROS levels are significantly increased in the (p)ppGpp^0^ and *rel*_*syn*_ mutants when grown in RPMI medium. Complementation of the (p)ppGpp^0^ mutant with the Rel synthetase on plasmid pCG327 resulted in ROS decrease (Fig. 5D). Of note, the ppGpp^0^ mutant showed a similar internal ROS level as the *katA* mutant, which is deficient in H_2_O_2_ detoxification and was used as positive control.

In addition, the FOX assay was used to determine the activities for detoxification of external H_2_O_2_ in cell extracts. The results revealed fast H_2_O_2_ detoxification within 4 min in stationary phase WT cells, but a delayed removal of external H_2_O_2_ in (p)ppGpp^0^ mutant cells (Fig. 5E). This points to an increased endogenous ROS level in the (p)ppGpp^0^ mutant, exceeding the ROS detoxification capacity of antioxidant enzymes. To exclude that the slower H_2_O_2_ detoxification ability in the (p)ppGpp^0^ mutant is caused by decreased translation of KatA, the *S. aureus* cell extracts were subjected to native PAGE and diaminobenzidine staining assay for visualization of catalase activity [76,77]. However, similar strong KatA activities were observed in WT and (p)ppGpp^0^ mutant cells (Fig. 5F), indicating that increased endogenous ROS levels in the (p)ppGpp^0^ mutant must overwhelm the antioxidant systems causing the delay in external H_2_O_2_ detoxification in the FOX assay (Fig. 5E). Together, our results confirm the hypothesis of higher ROS levels in the absence of (p)ppGpp. Increased ROS disturb the cellular redox balance, leading to an increased expression of the antioxidant response in the transcriptome.

### 3.5 Respiratory chain activity is elevated in the (p)ppGpp^0^ mutant

**Fig. 6:**
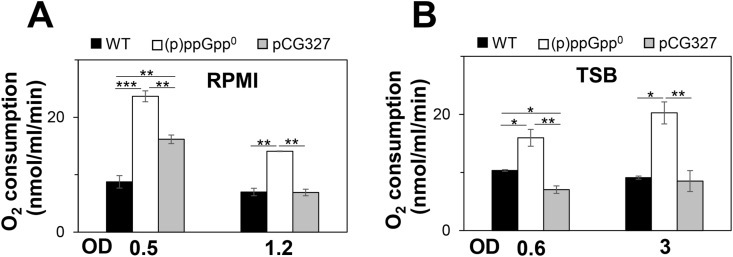
**The (p)ppGpp**^**0**^**mutant exhibits an elevated respiratory chain activity, contributing to ROS formation. (A,B)** The *S. aureus* USA300JE2 WT, (p)ppGpp^0^ mutant and pCG327 complemented strain were grown in RPMI **(A)** and TSB **(B)** and harvested at an OD_500_ of 0.5 and 1.2 in RPMI and at an OD_600_ of 0.6 and 3 in TSB. The oxygen consumption rates were determined with 1 mM glucose as electron donor using the Clark-type electrode in three biological replicates. Error bars represent the standard deviation. *p < 0.05; **p < 0.01; ***p < 0.001.

Transcription of TCA cycle enzymes (*citCZ* and *sucCD*) and the terminal oxidases (*cydAB*) was 2-6-fold enhanced in the (p)ppGpp^0^ mutant. Thus, we hypothesized that higher respiratory chain activity could contribute to elevated ROS levels in the (p)ppGpp^0^ mutant. Oxygen consumption was measured in the WT, (p)ppGpp^0^ mutant and pCG327 complemented strain in RPMI and TSB medium with 1 mM glucose as electron donor (Fig. 6A and B). Indeed, the (p)ppGpp^0^ mutant showed significantly 1.5-3-fold increased oxygen reduction rates compared to the WT and complemented strain during the log and stationary phases in RPMI and TSB medium (Fig. 6A and B). These results confirmed that respiratory chain activity is elevated in the absence of (p)ppGpp, leading to enhanced ROS production.

### 3.6 The (p)ppGpp^0^ mutant is susceptible to iron excess due to elevated total intracellular iron levels, including the labile iron pool

**Fig. 7:**
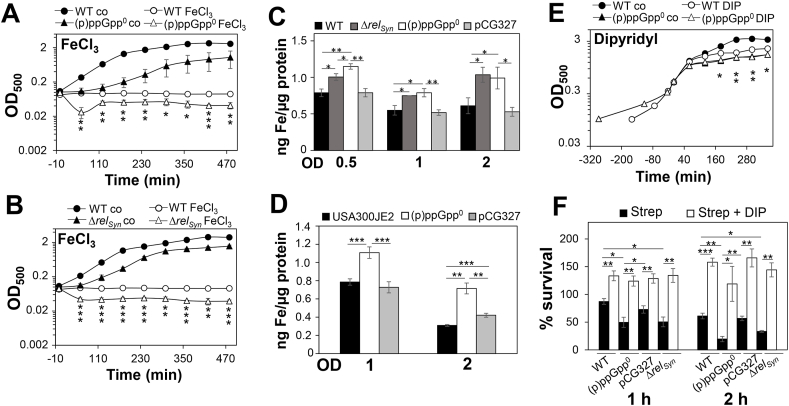
**The (p)ppGpp**^**0**^**mutant is sensitive to iron excess due to increased intracellular total and free iron levels. (A, B)** The growth curves of the *S. aureus* USA300JE2 WT, (p)ppGpp^0^ and *rel*_*syn*_ mutants were monitored in RPMI with and without 120 μM FeCl_3_. **(C, D)** The total cellular iron levels were determined with ferene-s assay **(C)** and ICP-MS analysis **(D)**. Total intracellular iron levels are significantly increased in the (p)ppGpp^0^ and *rel*_*syn*_ mutants as compared to the WT and the complemented strain pCG327 during the log and stationary phases. **(E)** The (p)ppGpp^0^ mutant was not impaired in growth after addition of 10 mM dipyridyl (DIP) at an OD_500_ of 0.5 as compared to the WT. **(F)** In addition, survival assays of *S. aureus* strains were performed after 1 and 2 h of treatment with the antibiotic streptonigrin at an OD_500_ of 0.5 in the presence or absence of dipyridyl (DIP). The (p)ppGpp^0^ and *rel*_*syn*_ mutants showed increased sensitivity to streptonigrin, indicating an elevated labile iron pool. The addition of DIP restored the survival of all strains. The results are from 3 to 4 biological replicates. Error bars represent the standard deviation. Statistical tests in A, B and E are shown for “WT FeCl_3_/DIP” vs. “ppGpp^0^/Δ*rel*_*syn*_ FeCl_3_/DIP “: *p < 0.05; **p < 0.01; ***p < 0.001.

The transcriptome analysis revealed an oxidative and iron stress response with the highest fold-changes for *dps* and *ftnA* encoding iron storage ferritins in the (p)ppGpp^0^ mutant in RPMI during the stationary phase (Fig. 2, Tables S4–S5). This could point to higher iron levels in the (p)ppGpp^0^ mutant, which might be responsible for the growth delay in RPMI (Fig. 3C). Growth in RPMI medium was shown to mimic infection conditions of *S. aureus* in human plasma resulting in higher expression levels of iron-regulated genes [92]. To test the hypothesis of increased endogenous iron levels, we analyzed the growth of the (p)ppGpp^0^ and *rel*_*syn*_ mutants under iron excess with 120 μM FeCl_3_. The (p)ppGpp^0^ and *rel*_*syn*_ mutants were both more sensitive in growth under iron excess relative to the WT (Fig. 7A and B), which could point to internal iron overload.

Thus, the total intracellular iron levels of the *S. aureus* WT, (p)ppGpp^0^ and *rel*_*syn*_ mutants as well as the Rel_Syn_ complemented strains were determined during the log and stationary phases using ferene-s assay and ICP-MS analysis (Fig. 7C and D). In agreement with our hypothesis, the (p)ppGpp^0^ mutant showed significantly increased internal total iron levels during the log and stationary phases. The iron concentrations could be reversed to WT level in the Rel_syn_ complemented strain. In addition, the (p)ppGpp^0^ mutant was not impaired in growth under iron starvation with dipyridyl in contrast to the WT, further supporting that the (p)ppGpp^0^ mutant suffers from internal iron overload (Fig. 7E).

Previous studies showed that increased ROS levels destroy FeS clusters leading to the release of free iron as labile iron pool [93,94]. To determine whether the increased total iron level in the (p)ppGpp^0^ mutant is caused by an elevated labile iron pool, we determined the sensitivity of the strains to the aminoquinone antibiotic streptonigrin using survival assays. The toxic effect of streptonigrin involves DNA damage by complexing the labile iron pool and autoxidation leading to ROS formation [95]. In fact, both (p)ppGpp^0^ and *rel*_*syn*_ mutants showed 1.7-3-fold decreased survival after streptonigrin intoxication compared to the WT and pCG327 complemented strain, supporting that higher free iron levels accumulate in the (p)ppGpp^0^ mutant in RPMI (Fig. 7F). To verify that the streptonigrin sensitivity depends on the internal iron level, *S. aureus* strains were exposed to dipyridyl prior to streptonigrin treatment. The survival of the (p)ppGpp^0^ and *rel*_*syn*_ mutants was strongly improved and restored to WT level after dipyridyl pre-treatment (Fig. 7F). These results indicate that FeS-cluster damage by ROS might contribute to the elevated total and free iron level in the (p)ppGpp^0^ mutant. Together, our data suggest that physiological (p)ppGpp levels lead to reduction of cellular free iron levels due to decreased respiratory chain activity in *S. aureus* during the stationary phase to prevent ROS generation and ensure long-term survival.

### 3.7 Elevated free iron levels contribute to ROS production and an oxidative stress response in the (p)ppGpp^0^ mutant during the stationary phase

**Fig. 8:**
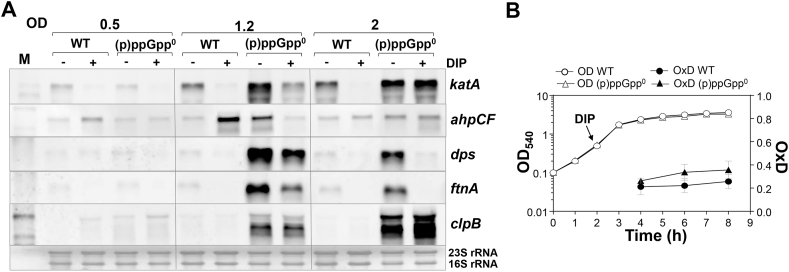
**Increased free cellular iron levels induce an oxidative stress response in the (p)ppGpp**^**0**^**mutant. (A)** Northern blot analysis was used to analyze transcription of PerR and CtsR regulon genes in *S. aureus* USA300JE2 WT and the (p)ppGpp^0^ mutant in RPMI medium with and without 10 mM dipyridyl (DIP), which was added at an OD_500_ of 0.5. Dipyridyl partially decreased transcription of genes for iron storage (*dps* and *ftnA*) and H_2_O_2_ detoxification (*katA*, *ahpCF*) in the (p)ppGpp^0^ mutant. The methylene blue stain is the RNA loading control indicating the bands of the 16S and 23S rRNAs. **(B)** The basal level of *E*_BSH_ was determined using Brx-roGFP2 biosensor along the growth curve in *S. aureus* USA300JE2 WT and the (p)ppGpp^0^ mutant after addition of dipyridyl.

To investigate whether elevated free iron levels induce ROS formation and the oxidative stress response in the (p)ppGpp^0^ mutant, transcription of PerR regulon genes was analyzed after dipyridyl addition (Fig. 8A). The Northern blot results showed that dipyridyl decreased transcription of the antioxidant genes *ahpCF* and *katA* during the stationary phase at an OD_500_ of 1.2, but not at an OD_500_ of 2. In addition, transcription of genes encoding iron storage ferritins *dps* and *ftnA* was decreased by dipyridyl in the (p)ppGpp^0^ mutant, especially during the later stationary phase (Fig. 8A). However, no difference in transcription was observed for the CtsR-regulated *clpB* gene after dipyridyl addition. These results support that elevated iron levels in the (p)ppGpp^0^ mutant partly lead to the induction of the oxidative and iron stress response as a consequence of Fenton-induced ROS formation. To verify that iron-induced ROS levels cause disturbance of the cellular redox potential in the (p)ppGpp^0^ mutant, we monitored the *E*_BSH_ changes using the Brx-roGFP2 biosensor along the growth in the presence of dipyridyl. The addition of dipyridyl resulted in a reductive shift of *E*_BSH_ in the (p)ppGpp^0^ mutant, with no significant difference to the dipyridyl-treated WT (Fig. 8B, Table S3). These data support that iron-induced ROS formation results in an impaired redox balance in the absence of (p)ppGpp.

### 3.8 (p)ppGpp confers tolerance to HOCl stress during the stationary phase by limiting endogenous ROS formation

**Fig. 9:**
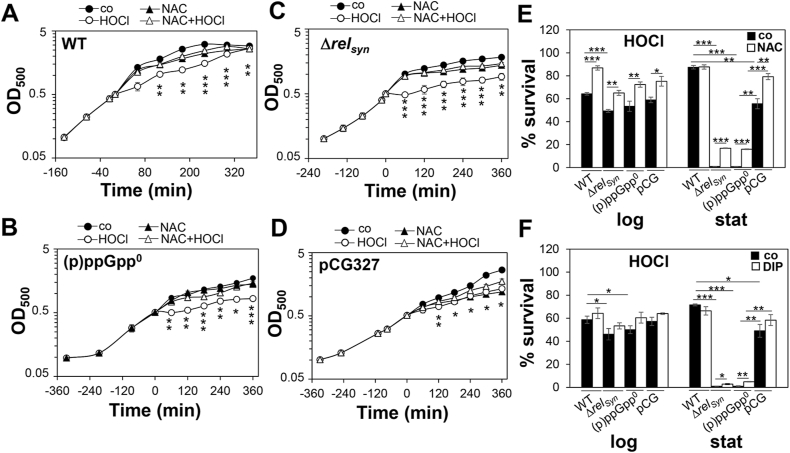
**ROS and iron scavengers protect the (p)ppGpp**^**0**^**mutant against oxidative stress. (A**–**D)** For the growth curves, *S. aureus* USA300JE2 WT, (p)ppGpp^0^ and Δ*rel*_*syn*_ mutants as well as the complemented strain (pCG327) were grown in RPMI until an OD_500_ of 0.5 and treated with sub-lethal concentrations of 1.25 mM N-acetyl cysteine (NAC) and 1.5 mM HOCl. **(E, F)** Survival assays were performed by treatment of the *S. aureus* strains with 3.5 mM HOCl and 1.25 mM N-acetyl cysteine (NAC) **(E)** or 10 mM dipyridyl (DIP) **(F)** at OD_500_ of 0.5 and 2. The CFUs were determined after 1 h stress exposure and survival rates calculated relative to the control, which was set to 100%. The addition of N-acetyl cysteine and dipyridyl significantly improved the resistance against HOCl of the (p)ppGpp^0^ and *rel*_*syn*_ mutants. The HOCl sensitivity of the stationary phase (p)ppGpp^0^ mutant could be restored partially to wild-type levels in the pCG327 complemented strain. The results are from three biological replicates. Error bars represent the standard deviation. Statistical test for the growth curves in A–D: “HOCl” vs “NAC + HOCl”: *p < 0.05; **p < 0.01; ***p < 0.001.

We hypothesized that enhanced ROS levels contribute to the impaired redox balance in the (p)ppGpp^0^ mutant, which confers the HOCl-sensitive phenotype during the stationary phase. To investigate the role of iron-induced ROS in terms of HOCl susceptibility, growth and survival assays were performed with the ROS scavenger N-acetyl cysteine added prior to HOCl exposure. The (p)ppGpp^0^ and *rel*_*syn*_ mutants were strongly impaired in growth and survival under HOCl stress without ROS scavengers during stationary phase (Fig. 9A–F). Pre-treatment with N-acetyl cysteine strongly improved the growth and survival of stationary phase (p)ppGpp^0^ and *rel*_*syn*_ mutants under HOCl stress (Fig. 9A–E). The protective effect of dipyridyl in HOCl stress survival was less significant in the mutants (Fig. 9F). While survival of stationary phase (p)ppGpp^0^ and *rel*_*syn*_ mutants under HOCl was increased by 20% with N-acetyl cysteine, dipyridyl-exposed mutant cells showed only 2–5% elevated viability (Fig. 9E and F). However, while growth of the (p)ppGpp^0^ and *rel*_*syn*_ mutants could be fully restored with N-acetyl cysteine, the survival could not be fully restored to WT level (Fig. 9A–E). These results indicate that the HOCl susceptibility of the (p)ppGpp^0^ and *rel*_*syn*_ mutants is caused by ROS formation, which can be limited by ROS scavengers. Apart from the reduction of iron and ROS levels, additional mechanisms account for the increased tolerance of *S. aureus* towards HOCl stress by (p)ppGpp during the stationary phase.

### 3.9 (p)ppGpp contributes to antibiotics tolerance towards ciprofloxacin and tetracycline during the log and stationary phase by reduction of iron-induced ROS formation

**Fig. 10:**
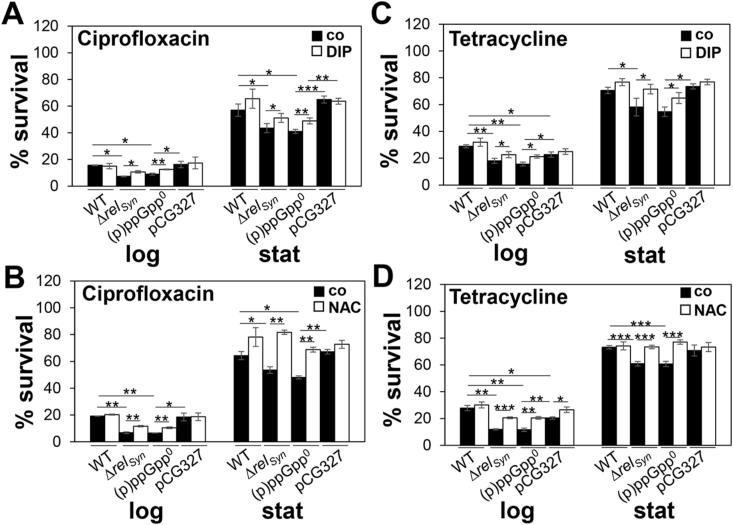
**ROS and iron scavengers enhance survival of the (p)ppGpp**^**0**^**and Δ*rel***_***syn***_**mutants under ciprofloxacin and tetracycline stress. (A**–**D)** For survival assays, *S. aureus* USA300JE2 WT, (p)ppGpp^0^ and Δ*rel*_*syn*_ mutants as well as the complemented strain (pCG327) were grown in RPMI until an OD_500_ of 0.5 and 2 for log and stationary phase. Cells were treated with 90.5 μM ciprofloxacin **(A,B)** or 62.38 mM tetracycline **(C,D)** in the presence or absence of 10 mM dipyridyl (DIP) **(A,C)** or 1.25 mM N-acetyl cysteine (NAC) **(B,D)**, respectively. The CFUs were determined after 2 h stress exposure and survival rates calculated relative to the untreated control, which was set to 100%. The addition of N-acetyl cysteine and dipyridyl significantly improved the survival of the (p)ppGpp^0^ and Δ*rel*_*syn*_ mutants under antibiotics stress. The results are from 3 to 4 biological replicates. Error bars represent the standard deviation. *p < 0.05; **p < 0.01; ***p < 0.001.

The alarmone (p)ppGpp has been shown to contribute to the tolerance to various antibiotics in *S. aureus*, including vancomycin, ampicillin and other β-lactam antibiotics [47,48,51,52]. Due to the involvement of ROS in the killing mode of different antibiotic classes [59,96], we were interested whether (p)ppGpp confers tolerance to antibiotics by limiting ROS formation during the stationary phase. In survival assays, we could confirm that the *S. aureus* USA300JE2 WT acquires a 2–3 fold increased tolerance to the antibiotics ciprofloxacin and tetracycline during the stationary phase (Fig. 10). Both (p)ppGpp^0^ and *rel*_*syn*_ mutants are more sensitive in growth to sub-lethal concentrations of 5.19 mM tetracycline and 90.5 μM ciprofloxacin as compared to the WT (Figs. S4 and S5). In addition, both mutants displayed a 5–15% decreased survival after exposure to 90.5 μM ciprofloxacin and 62.38 mM tetracycline during the log and stationary phases relative to its parent (Fig. 10). The growth and survival phenotype of the (p)ppGpp^0^ mutant could be restored in the (p)ppGpp complemented strain (Fig. 10, Fig. S4DH and Fig. S5DH). Treatment of the (p)ppGpp^0^ and *rel*_*syn*_ mutants with N-acetyl cysteine or dipyridyl prior to antibiotics exposure significantly improved the growth and survival and restored their tolerance to the antibiotics to WT levels (Fig. 10, Fig. S4BCFG and Fig. S5BCFG). Of note, the protection of the (p)ppGpp^0^ mutant against ciprofloxacin-induced ROS formation was stronger with ROS scavengers, while dipyridyl showed a smaller protective effect (Fig. 10A,B). In addition, the (p)ppGpp^0^ mutant was still able to acquire an enhanced tolerance to antibiotics during the stationary phase (Fig. 10), indicating that other stationary phase mechanisms must contribute to antibiotics tolerance. Taken together, our results support that (p)ppGpp contributes to oxidative stress protection and antibiotics tolerance in *S. aureus* during the stationary phase by reducing cellular free iron-levels and aerobic respiration to limit ROS formation and to regenerate redox homeostasis.

## 4 Discussion

In this study, we have shown that *S. aureus* cells can acquire an improved tolerance towards HOCl and MHQ during the stationary phase, which was dependent on the small alarmone (p)ppGpp. Transcriptome analyses of stationary phase (p)ppGpp^0^ mutant cells revealed high expression of genes for iron-storage ferritins and miniferritin (*dps, ftnA*) as well as the induction of the PerR, QsrR, CstR, CtsR and HrcA regulons, indicating an oxidative and iron stress response. Of note, this starvation-induced oxidative stress response was only observed in cell culture RPMI medium, resulting in a growth delay of the (p)ppGpp^0^ mutant. We anticipate that the oxidative stress phenotype of the (p)ppGpp^0^ mutant in RPMI is related to its lower amounts of ROS scavenging components, retaining higher levels of oxidizing equivalents of HOCl and H_2_O_2_ [97]. In contrast, nutrient-rich LB and TSB contain high amounts of ROS-quenching amino acids, peptides and the antioxidant tripeptide glutathione (GSH), which scavenge ROS and thereby decrease oxidant toxicity [97]. Since RPMI resembles infection conditions in human plasma [92], this could be relevant for long-term and chronic *S. aureus* infections and highlights the crucial role of (p)ppGpp for survival of starved bacteria [52,98].

Based on the transcriptome signature, we hypothesized that ROS levels are increased in the absence of (p)ppGpp during the stationary phase. Indeed, the (p)ppGpp^0^ mutant revealed a slightly impaired redox state, higher endogenous ROS levels and apparently overloaded antioxidant enzymes, which were delayed in H_2_O_2_ detoxification. ROS increase in the (p)ppGpp^0^ mutant could be attributed to elevated intracellular iron levels and higher respiratory chain activities, resulting in an oxidative and iron stress response. In support of iron-induced ROS formation, the PerR-dependent oxidative stress response was partly abolished by the iron chelator dipyridyl. Moreover, the impaired redox balance and iron excess are responsible for the susceptibility of the (p)ppGpp^0^ mutant towards HOCl stress, since ROS and iron scavengers improved the growth and survival of the mutant.

Elevated iron levels in the (p)ppGpp^0^ mutant resulted in induction of iron storage ferritins and strong Fur-dependent repression of uptake systems for iron, heme and siderophores to prevent further iron intoxication. Increased ROS and iron levels were previously shown to induce the PerR regulon in *S. aureus*, supporting the connection between iron excess and oxidative stress [23,99]. However, it seems counterintuitive, that despite the growth-limiting effects of increased iron levels, FeS cluster synthesis is still increased in the (p)ppGpp^0^ mutant. The enhanced need for FeS cluster synthesis might be explained by ROS poisoning of exposed FeS clusters of dehydratases, such as the TCA cycle enzymes aconitase and fumarase or the isopropylmalate dehydratase LeuCD [100,101]. This is supported by an elevated transcription of TCA cycle genes in the absence of (p)ppGpp. The release of free iron from oxidized FeS-clusters [93,94] could be responsible for elevated endogenous iron levels, potentiating ROS formation in the (p)ppGpp^0^ mutant. In agreement with this hypothesis, the (p)ppGpp^0^ mutant showed an increased pool of labile iron as indicated by its sensitivity to the antibiotic streptonigrin. Thus, respiratory ROS increase might be the first event leading to an increased internal iron level that further potentiates ROS formation through the Fenton chemistry.

Strikingly, we previously showed that overproduction of (p)ppGpp during the log phase also results in the activation of genes involved in oxidative stress and iron-storage (*ftnA, dps*) independently of the global regulators PerR, Fur, SarA or CodY [60]. We hypothesize that the iron and oxidative stress response induced by (p)ppGpp during the log phase protects *S. aureus* from anticipated future stress and to inhibit toxic iron accumulation, whereas the (p)ppGpp^0^ mutant responds to ROS increase due to elevated respiration via the classical PerR-dependent oxidative stress response. These findings suggest that physiological (p)ppGpp levels are intimately linked to redox and iron homeostasis of the cells.

**Fig. 11:**
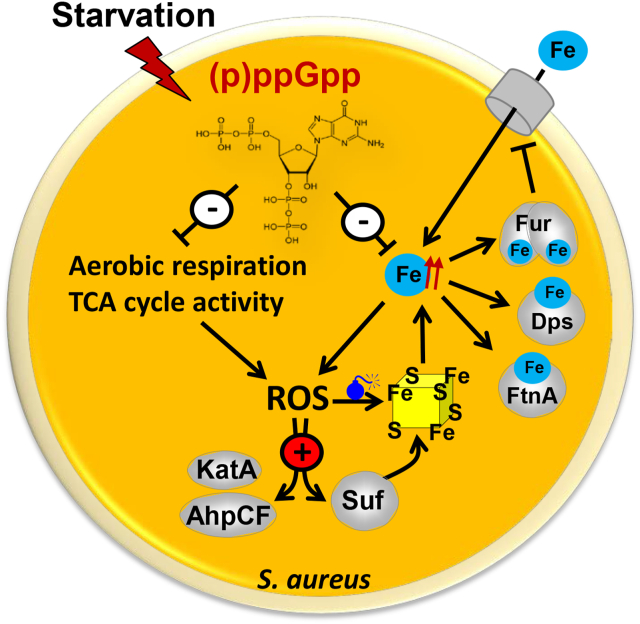
**Schematics of (p)ppGpp regulated tolerance to oxidants and antibiotics in *S. aureus*.** (p)ppGpp down-regulates internal iron levels and respiratory chain activity leading to decreased ROS levels. In the (p)ppGpp^0^ mutant, increased iron levels cause induction of iron storage proteins (Dps, FtnA) and Fur-mediated repression of iron-uptake systems. Increased ROS levels in the (p)ppGpp^0^ mutant cause derepression of the PerR-controlled antioxidant systems (KatA, AhpCF) for ROS detoxification and the Suf machinery for FeS-cluster biosynthesis, since ROS destroy FeS clusters potentiating the release of free iron and in turn ROS levels. These ROS and iron-mediated responses are repressed by (p)ppGpp leading to a reductive shift in *E*_BSH_, which promotes tolerance to HOCl and ROS produced by the antibiotics tetracycline and ciprofloxacin in *S. aureus*.

Similar connections between iron, aerobic respiratory chain activity, ROS and the stringent response have been demonstrated in other bacteria. In *E. coli,* (p)ppGpp accumulated in response to iron starvation, leading to induction of Fur-controlled iron uptake systems [102]. In this case, SpoT has been proposed to act as direct sensor for Fe^2+^ or Fe^3+^ inside the cell [102]. Similar to *S. aureus,* (p)ppGpp has been proposed to decrease iron and ROS levels in *V. cholerae*, which promotes tolerance to the antibiotic tetracycline [54]. Increased free iron levels were measured in the (p)ppGpp^0^ mutant in *V. cholerae*, which resulted in 10-fold elevated expression of the Fe(III) ABC transporter substrate-binding protein FbpA [54]. In contrast, (p)ppGpp accumulation led to repression of FbpA and reduced iron levels to prevent Fenton chemistry and ROS generation. Furthermore, expression of several TCA cycle enzymes, such as *acnB, icd, sucCD, sdhABC* and *mdh* was increased in the absence of (p)ppGpp suggesting enhanced TCA cycle activity and central carbon catabolism as another source of ROS in *V. cholerae* [54]. Similarly, transcription of *acnA (citB), citC, citZ, sucCD, sdhABCD* and *cydAB* was 2-4-fold enhanced in the *S. aureus* (p)ppGpp^0^ mutant, supporting higher TCA cycle and respiratory chain activity, leading to increased ROS levels. Thus, in *S. aureus* the induction of the SR represses respiratory chain activity and endogenous iron levels to lower ROS levels, contributing to the tolerance towards oxidative stress and antibiotics (Fig. 11).

In *Pseudomonas aeruginosa*, decreased activities of the antioxidant enzymes catalase and superoxide dismutase have been determined in the (p)ppGpp^0^ mutant, leading to decreased detoxification of superoxide anion and H_2_O_2_ [55,56]. Increased ROS levels in SR mutants have been associated with enhanced susceptibility to H_2_O_2_ stress and antibiotics during the stationary phase [55,56]. Similarly, the *S. aureus* (p)ppGpp^0^ mutant was more susceptible to H_2_O_2_ [60] and HOCl stress during the stationary phase. While the *S. aureus* (p)ppGpp^0^ mutant showed decreased H_2_O_2_ detoxification ability, the catalase activity was not affected. Instead, the antioxidant systems seem to be busy with removal of internal ROS in the (p)ppGpp^0^ mutant, resulting in delayed detoxification of external H_2_O_2_. The (p)ppGpp^0^ mutant further showed a slight oxidized shift in the *E*_BSH_, explaining its susceptibility to survive HOCl stress exposure during the stationary phase.

Another study showed a protective effect of (p)ppGpp under nitrosative (NO) stress in the intestinal pathogen *Salmonella* Typhimurium [103]. Specifically, (p)ppGpp was shown to activate transcription of biosynthesis genes for branched chain amino acids to restore translation of flavohemoglobin Hmp, which is involved in NO detoxification [103]. Altogether, (p)ppGpp contributes in bacteria to ROS and RNS tolerance via different mechanisms, affecting iron and redox homeostasis, ROS levels, activities of antioxidant enzymes, central carbon catabolism and amino acid biosynthesis to facilitate translation of antioxidant and anti-nitrosative defense mechanisms.

In addition, we found that the *S. aureus* (p)ppGpp^0^ mutant is more susceptible to ROS generated by the antibiotics tetracycline and ciprofloxacin. The involvement of ROS in the bactericidal mode of action of various antibiotic classes is well established [59,61,96,104]. Our results revealed that ROS and iron scavengers increased the tolerance of the (p)ppGpp^0^ mutant towards tetracycline and ciprofloxacin, supporting that ROS and iron contributed to the antibiotics susceptibility. Thus, (p)ppGpp promotes tolerance to ROS-producing antibiotics by regulation of iron and redox homeostasis in *S. aureus*.

Altogether, we propose a model that (p)ppGpp down-regulates respiratory chain activity and free iron levels in *S. aureus*, leading to decreased internal ROS levels, which renders the cells tolerant to oxidative stress and antibiotics during the stationary phase (Fig. 11). Future studies will be directed to elucidate the molecular mechanisms of how (p)ppGpp modulates iron and redox homeostasis in *S. aureus*.

## Declaration of competing interest

No competing financial interests exist.

## List of abbreviations

BSH: bacillithiol
DTT: dithiothreitol
H_2_O_2_: hydrogen peroxide
HOCl: hypochlorous acid
LB: Luria Bertani
MHQ: methylhydroquinone
MRSA: methicillin-resistant *Staphylococcus aureus*
(p)ppGpp: guanosine tetra- and pentaphosphate
OD_500_: optical density at 500 nm
RCS: reactive chlorine species
RES: reactive electrophilic species
ROS: reactive oxygen species
SR: stringent response
