# Supplementary material for: The alarmone (p)ppGpp confers tolerance to oxidative stress during the stationary phase by maintenance of redox and iron homeostasis in Staphylococcus aureus
Source: Free Radic Biol Med. 2020 Dec;161:351–64. doi: 10.1016/j.freeradbiomed.2020.10.322 (PMC7754856; doi:10.1016/j.freeradbiomed.2020.10.322)
Supplement: Multimedia component 1 [file mmc1.pdf]

Figure S1

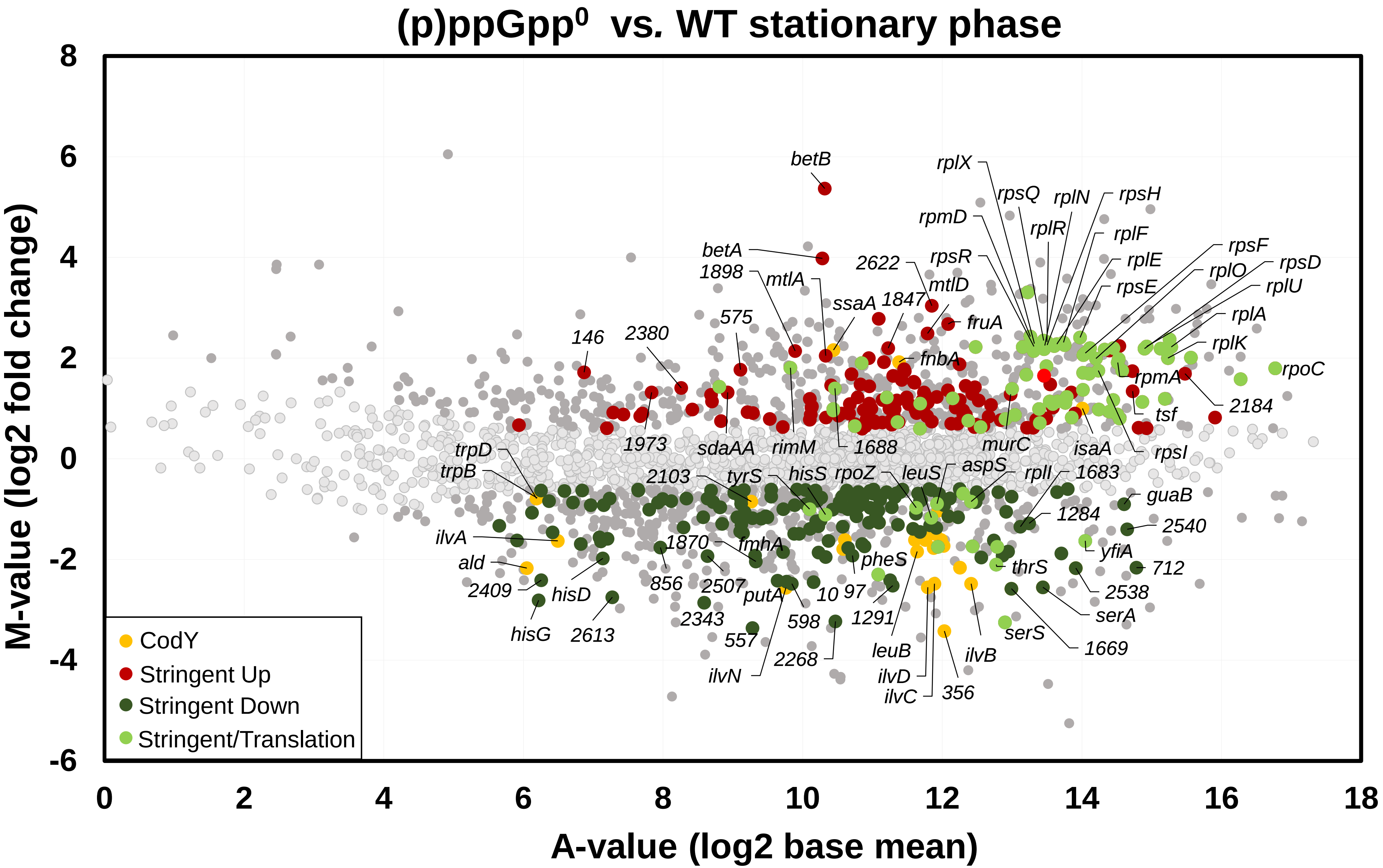

Fig. S1. Increased transcription of genes for ribosomal proteins in the (p)ppGpp^0 mutant is revealed by transcriptome analysis. *S. aureus* USA300JE2 and the (p)ppGpp^0 mutant were grown in RPMI and RNA was isolated from cells harvested at an OD_{500} at 1.2. The gene expression profile of the (p)ppGpp^0 mutant versus the WT is shown as ratio/intensity scatterplot (M/A-plot), which is based on the differential gene expression analysis using DeSeq2. Colored symbols indicate significantly induced and repressed transcripts (M-value ≥0.6 or ≤−0.6; p ≤ 0.05. Light gray symbols denote transcripts with no fold-changes (p > 0.05). Genes were assigned to the functional category "Translation" based on the *S. aureus* USA300 FPR3757 public available annotation. The (p)ppGpp0 mutant shows an increased transcription of genes for ribosomal proteins involved in translation. The complete transcriptome data and regulon classifications are listed in Tables S4-S6.

Figure S2

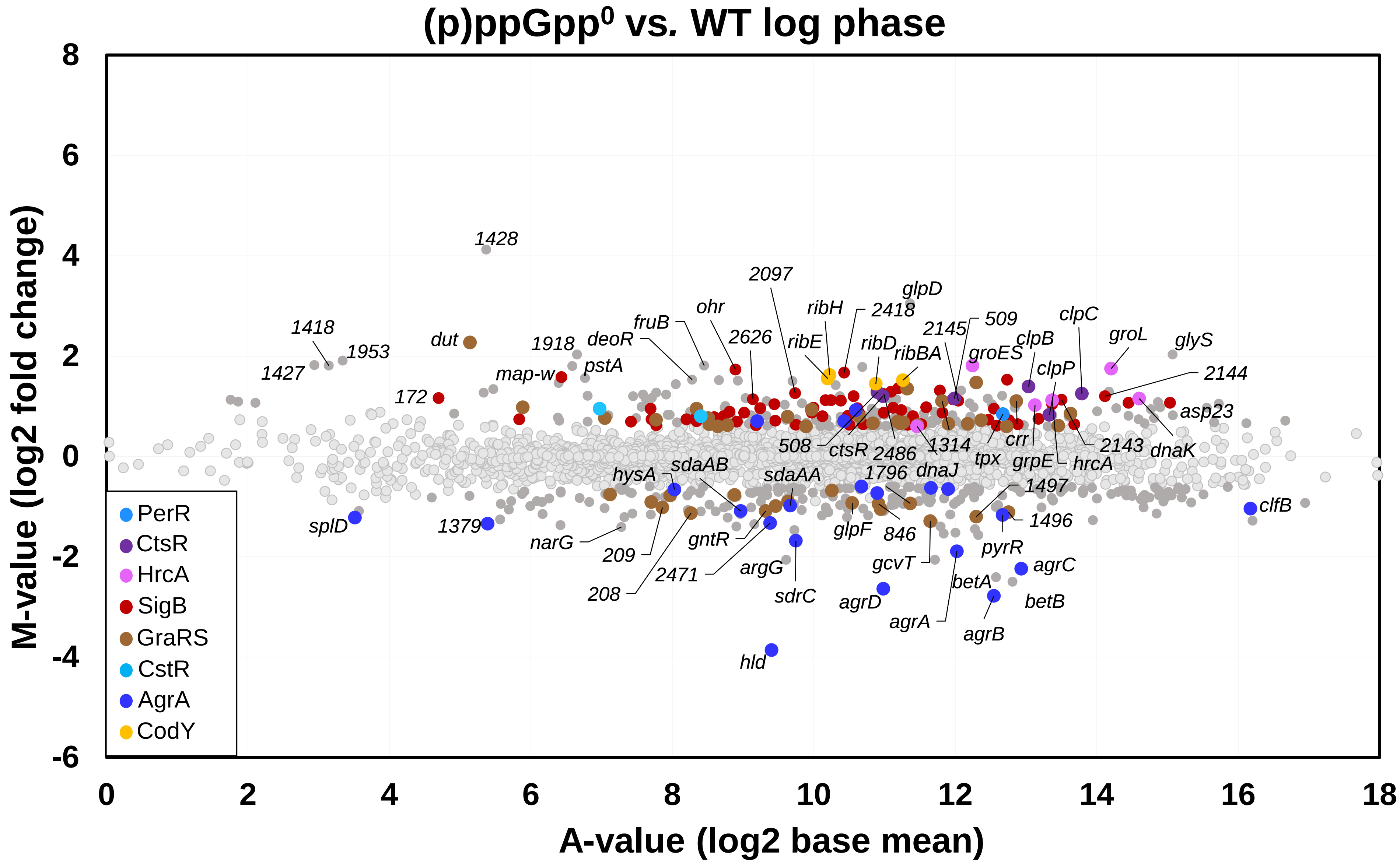

Fig. S2. Transcriptome analysis of the *S. aureus* USA300JE2 (p)ppGpp^0 mutant versus the WT in RPMI medium during the log phase. *S. aureus* USA300JE2 and the (p)ppGpp^0 mutant were grown in RPMI medium until an OD_{500} of 0.5 and harvested for RNA isolation. The gene expression profile of the (p)ppGpp^0 mutant in comparison to the WT is shown as ratio/intensity scatterplot (M/A-plot), which is based on the differential gene expression analysis using DeSeq2. Colored symbols indicate significantly induced and repressed transcripts (*M*-value ≥0.6 or ≤−0.6; $p \leq 0.05$). The CodY (yellow), CtsR (magenta) and HrcA (pink) regulons display only weak fold-changes in the (p)ppGpp^0 mutant compared to the WT. Light gray symbols denote transcripts with no fold-changes ($p > 0.05$). The complete transcriptome data and regulon classifications are listed in **Tables S7-S9**.

Figure S3

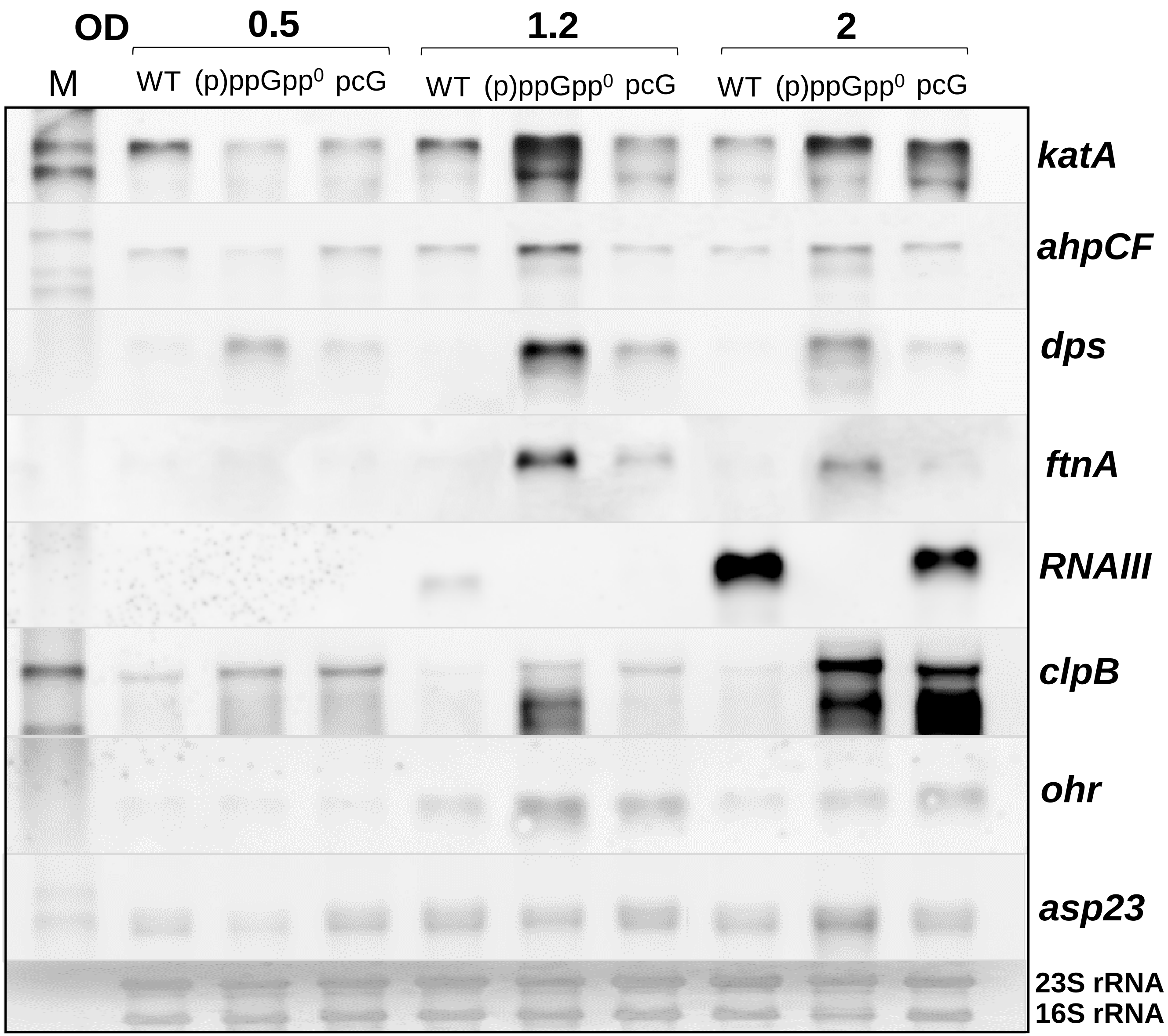

Fig. S3. Transcriptional induction of the oxidative and iron stress response in the (p)ppGpp^0 mutant is abolished in the *S. aureus* (p)ppGpp complemented strain (pCG327). RNA was isolated from the *S. aureus* USA300JE2 WT, (p)ppGpp^0 and the complemented strain (pCG327)mafter growth in RPMI medium during the log and stationary phase at OD_{500}=0.5, 1.2 and 2. Transcription of genes of the PerR (*katA, ahpCF, dps,* and *ftnA*), SigmaB (*asp23, ohr*), Agr (*RNAIII*) and CtsR (*clpB*) regulons was analyzed using Northern blots similar as in Fig.3. The methylene blue stain is the RNA loading control showing the abundant 16S and 23S rRNAs.

Figure S4

Ciprofloxacin

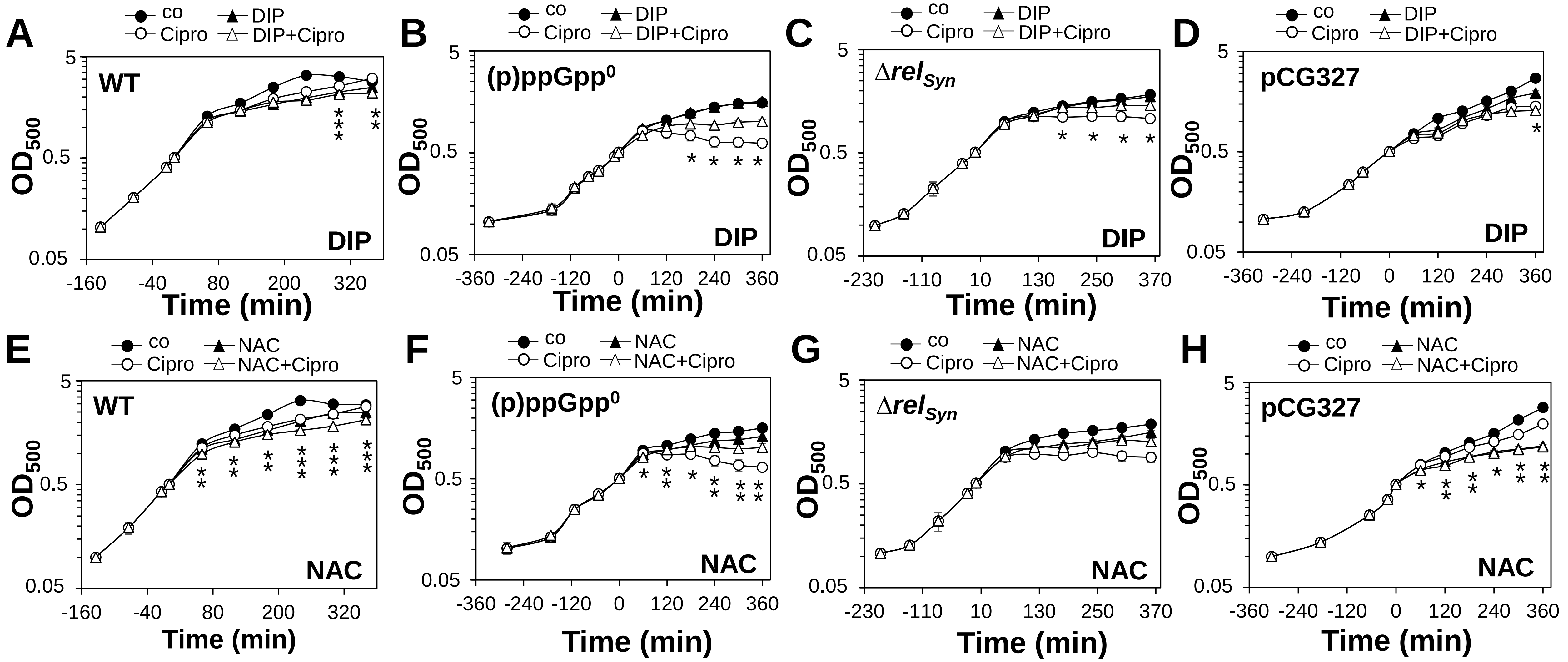

Fig. S4. Iron and ROS scavengers increase tolerance of the (p)ppGpp^0 and *rel*$_{Syn}$ mutant after ciprofloxacin stress. (A-H) For the growth curves, *S. aureus* USA300JE2 wild type, (p)ppGpp^0 and Δ*rel*$_{Syn}$ mutants as well as the complemented strain (pCG327) were grown in RPMI medium until an OD_{500} of 0.5 and treated with sub-lethal concentration of 10 mM dipyridyl (DIP) **(A-D)** or 1.25 mM N-acetyl cysteine (NAC) **(E-H)** prior to exposure of 90.5 µM ciprofloxacin. The addition of dipyridyl and N-acetyl cysteine significantly improves the growth of the (p)ppGpp^0 and *rel*$_{Syn}$ mutants under ciprofloxacin stress. The results are from 3-4 biological replicates. Error bars represent the standard deviation. Statistical test “Cipro“ vs “NAC/DIP+Cipro“: *$p < 0.05$; **$p < 0.01$; ***$p < 0.001$.

Figure S5

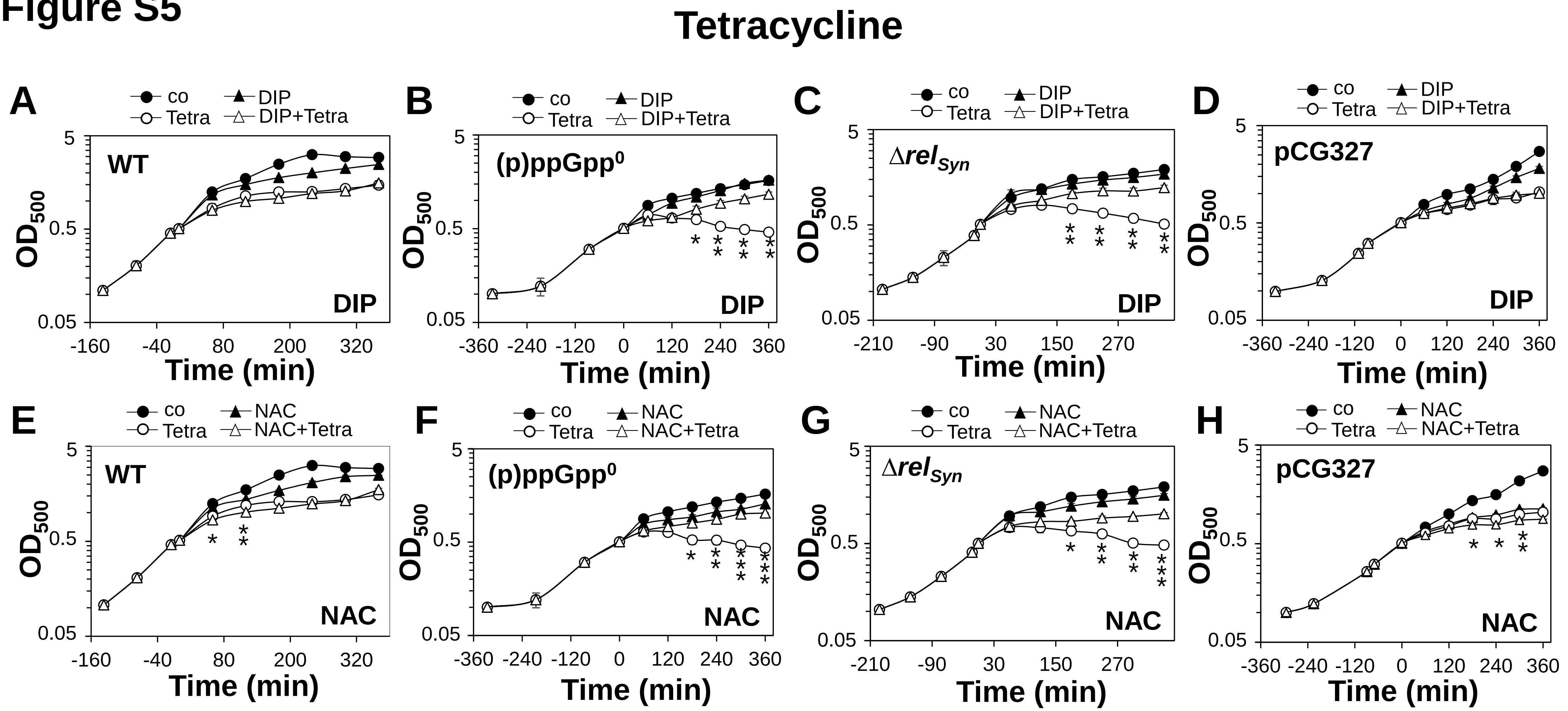

Fig. S5. Iron and ROS scavengers increase the tolerance of the (p)ppGpp⁰ and *rel*$_{Syn}$ mutant after tetracycline treatment. For the growth curves, *S. aureus* USA300JE2 WT, (p)ppGpp⁰ and Δ*rel*$_{Syn}$ mutants as well as the complemented strain (pCG327) were grown in RPMI until an OD_{500} of 0.5 and treated with sub-lethal concentrations of 10 mM dipyridyl (DIP) **(A-D)** or 1.25 mM N-acetyl cysteine (NAC) **(E-H)** prior to exposure of 5.19 mM tetracycline**.** The addition of N-acetyl cysteine and dipyridyl significantly improves the growth of the (p)ppGpp⁰ and *rel*$_{Syn}$ mutants under tetracycline stress. The results are from 3-4 biological replicates. Error bars represent the standard deviation. Statistical test "Tetra" vs "NAC/DIP+Tetra": *$p < 0.05$; **$p < 0.01$; ***$p < 0.001$.
