# Supplementary material for: The alarmone (p)ppGpp confers tolerance to oxidative stress during the stationary phase by maintenance of redox and iron homeostasis in Staphylococcus aureus
Source: Free Radic Biol Med. 2020 Dec;161:351–64. doi: 10.1016/j.freeradbiomed.2020.10.322 (PMC7754856; doi:10.1016/j.freeradbiomed.2020.10.322)
Supplement: Multimedia component 2 [file mmc2.pdf]

Table S1. Bacterial strains and plasmids

Strain	Description	Reference
Escherichia coli		
DH5α	F-φ80dlacZ Δ(lacZYA-argF) U169 deoRsupE44ΔlacU169 (f80lacZDM15) hsdR17 recA1 endA1 (rk- mk+) supE44gyrA96 thi-1 gyrA69 relA1	[1]
Staphylococcus aureus		
COL	archaic HA-MRSA strain	[2]
RN4220	restriction negative MSSA cloning intermediate derived from 8325-4	[3]
USA300JE2	MRSA strain with cured plasmids	[4]
USA300JE2 (p)ppGpp^0	USA300JE2 *relP, relQ, rel* active site mutant	[5]
USA300JE2 Δ*rel*$_{Syn}$	USA300JE2 *rel* synthetase domain mutant	[6]
USA300JE2 ppGpp^0::pCG327	USA300JE2 *relP, relQ, rel* active site mutant with AHT-inducible Rel synthetase	[7]
USA300JE2 *brx-roGFP2*	USA300JE2 pRB473-*brx-roGFP2*	This study
USA300JE2-ppGpp^0 *brx-roGFP2*	USA300JE2 *relP, relQ, rel* active site mutant expressing pRB473-*brx-roGFP2*	This study
COL Δ*katA*	COL *katA* deletion mutant	[8]
**plasmids**		
pRB473	pRB373-derivative, *E. coli S. aureus* shuttle vector, containing xylose-inducible P_{Xyl} promoter Amp^R, Cm^R	[9]
pRB473-*brx-roGFP2*	pRB473-derivative expressing *brx-roGFP2* under P_{Xyl}	[10]
pCG327	AHT inducible Rel synthetase with mutated N-terminus of hydrolase in pCG248	[7]

^{R}: resistant, Amp: ampicillin, Cm: chloramphenicol, AHT: anhydrotetracyclin

Table S2. Oligonucleotide sequences

Primer name	Sequence (5’ to 3’)
dps-for	TCAACAAGTAGCAAACTGGACA
dps-rev	CTAATACGACTCACTATAGGGAGACAATTATGTTTATCTACTGATGTTTGC
ohr-for	TGGCAATACATTATGAAACTAAAGC
ohr-rev	CTAATACGACTCACTATAGGGAGATTTAAATCGACATTAATATTTCCTTGA
katA-for	AAAGGTTCTGGTGCATTTGG
katA-rev	CTAATACGACTCACTATAGGGAGAAATGTGTTCCTCCACCTTGG
ahpC-for	TCCTGCTGACTTCTCATTCGT
ahpC-rev	CTAATACGACTCACTATAGGGAGAGGTTGCAATGTTTTAGCGCC
ftnA-for	TGAGTACTTTGCAGCACACG
ftnA-rev	CTAATACGACTCACTATAGGGAGAATTGCTGTCATCGCCGATAC
clpB-for	CCAAGCATGTGCAACAATTC
clpB-rev	CTAATACGACTCACTATAGGGAGACGTCAGTATGCGCTTTTTCA

Table S3. The BSH redox potential (E_{BSH}) in *S. aureus* USA300JE2 WT and the (p)ppGpp^0 mutant along the growth in LB - / + dipyridyl

Time (h)	E_{BSH} (mV)			
	- dipyridyl		+ dipyridyl	
	WT	(p)ppGpp^0	WT	(p)ppGpp^0
4	-291.8±2.9	-278.0±0.3	-299.3±5.8	-293.9±1.2
6	-287.0±0.6	-280.4±1.9	-298.1±5.3	-289.7±4.4
8	-290.0±1.3	-280.5±1.6	-297.2±3.8	-288.4±4.6

Supplementary References

[1] F.W. Studier, B.A. Moffatt, Use of bacteriophage-T7 RNA-polymerase to direct selective high-level expression of cloned genes, J Mol Biol 189(1) (1986) 113-130.

[2] W.M. Shafer, J.J. Iandolo, Genetics of staphylococcal enterotoxin B in methicillin-resistant isolates of *Staphylococcus aureus*, Infect Immun 25(3) (1979) 902-11.

[3] B.N. Kreiswirth, S. Lofdahl, M.J. Betley, M. O'Reilly, P.M. Schlievert, M.S. Bergdoll, R.P. Novick, The toxic shock syndrome exotoxin structural gene is not detectably transmitted by a prophage, Nature 305(5936) (1983) 709-12.

[4] P.D. Fey, J.L. Endres, V.K. Yajjala, T.J. Widhelm, R.J. Boissy, J.L. Bose, K.W. Bayles, A genetic resource for rapid and comprehensive phenotype screening of nonessential *Staphylococcus aureus* genes, mBio 4(1) (2013) e00537-12.

[5] P. Horvatek, A.M.F. Hanna, F.L. Gratani, D. Keinhörster, N. Korn, M. Borisova, C. Mayer, D. Rejman, U. Mäder, C. Wolz, Inducible expression of (p)ppGpp synthetases in *Staphylococcus aureus* is associated with activation of stress response genes, bioRxiv (2020) 2020.04.25.059725.

[6] T. Geiger, C. Goerke, M. Fritz, T. Schafer, K. Ohlsen, M. Liebeke, M. Lalk, C. Wolz, Role of the (p)ppGpp synthase RSH, a RelA/SpoT homolog, in stringent response and virulence of *Staphylococcus aureus*, Infect Immun 78(5) (2010) 1873-83.

[7] F.L. Gratani, P. Horvatek, T. Geiger, M. Borisova, C. Mayer, I. Grin, S. Wagner, W. Steinchen, G. Bange, A. Velic, B. Macek, C. Wolz, Regulation of the opposing (p)ppGpp synthetase and hydrolase activities in a bifunctional RelA/SpoT homologue from *Staphylococcus aureus*, PLoS Genet 14(7) (2018) e1007514.

[8] N. Linzner, V.N. Fritsch, T. Busche, Q.N. Tung, V.V. Loi, J. Bernhardt, J. Kalinowski, H. Antelmann, The plant-derived naphthoquinone lapachol causes an oxidative stress response in *Staphylococcus aureus*, Free Radic Biol Med 158 (2020) 126-136.

[9] R. Bruckner, E. Wagner, F. Götz, Characterization of a sucrase gene from *Staphylococcus xylosus*, J Bacteriol 175(3) (1993) 851-7.

[10] V.V. Loi, M. Harms, M. Müller, N.T.T. Huyen, C.J. Hamilton, F. Hochgräfe, J. Pane-Farre, H. Antelmann, Real-time imaging of the bacillithiol redox potential in the human pathogen *Staphylococcus aureus* using a genetically encoded bacilliredoxin-fused redox biosensor, Antioxid Redox Signal 26(15) (2017) 835-848.
